# Identification of candidate chemosensory genes in *Mythimna separata* by transcriptomic analysis

**DOI:** 10.1186/s12864-018-4898-0

**Published:** 2018-07-04

**Authors:** Lixiao Du, Xincheng Zhao, Xiangzhi Liang, Xiwu Gao, Yang Liu, Guirong Wang

**Affiliations:** 1 State Key Laboratory for Biology of Plant Diseases and Insect Pests, Institute of Plant Protection, Chinese Academy of Agricultural Sciences, Beijing, 100193 China; 2 Department of Entomology, College of Plant Protection, Henan Agricultural University, Zhengzhou, 450002 China; 3 Department of Entomology, China Agricultural University, Beijing, 100193 China

**Keywords:** *Mythimna separata*, Transcriptome, Chemosensory gene, Expression analysis

## Abstract

**Background:**

The oriental armyworm, *Mythimna separata*, is an economically important and common Lepidopteran pest of cereal crops. Chemoreception plays a key role in insect life, such as foraging, oviposition site selection, and mating partners. To better understand the chemosensory mechanisms in *M. separata*, transcriptomic analysis of antennae, labial palps, and proboscises were conducted using next-generation sequencing technology to identify members of the major chemosensory related genes.

**Results:**

In this study, 62 putative odorant receptors (OR), 20 ionotropic receptors (IR), 16 gustatory receptors (GR), 38 odorant binding proteins (OBP), 26 chemosensory proteins (CSP), and 2 sensory neuron membrane proteins (SNMP) were identified in *M. separata* by bioinformatics analysis. Phylogenetic analysis of these candidate proteins was performed. Differentially expressed genes (DEGs) analysis was used to determine the expressions of all candidate chemosensory genes and then the expression profiles of the three families of receptor genes were confirmed by real-time quantitative RT-PCR (qPCR).

**Conclusions:**

The important genes for chemoreception have now been identified in *M. separata*. This study will provide valuable information for further functional studies of chemoreception mechanisms in this important agricultural pest.

**Electronic supplementary material:**

The online version of this article (10.1186/s12864-018-4898-0) contains supplementary material, which is available to authorized users.

## Background

Insects live in environments where they are constantly surrounded by various chemical signals, including olfactory and taste. Perception of these chemical signals is crucial for insects, because they need to detect and distinguish these signals and then perform corresponding behaviors such as feeding, mating, oviposition, or escaping [1]. As the first step of chemosensory reception, chemical molecules are detected by sensory neurons housed within special chemosensory organs at the peripheral nerve level. Olfactory receptor neurons (ORNs) are distributed primarily on the antennae, and to a degree on the maxillary palps and labial palps. Gustatory receptor neurons (GRNs) are distributed on different taste organs over the entire body surface of insects including the proboscis, legs, wings, and even the female abdomen. Of these organs, the proboscis is the major gustatory organ in head [2].

The molecular mechanism of chemosensory reception of insects was determined in research focused on model insects, especially *Drosophila melanogaster*, in the last ten years. Many new gene families were discovered and their roles in chemosensory reception were established. At least three large and divergent receptor families (including odorant receptors (OR), ionotropic receptors (IR), and gustatory receptors (GR)), and three nonreceptor gene families (including odorant binding proteins (OBP), chemosensory proteins (CSP), and sensory neuron membrane proteins (SNMP)), are involved in the process of chemosensory reception [3–12].

Insect ORs, first identified in the *D. melanogaster* genome, contain seven transmembrane domains (TMDs) and a reversed membrane topology compared to vertebrate ORs [13, 14]. ORs are contained in the dendritic membrane of ORNs, and function as a heterodimer with a highly conserved non-canonical OR co-receptor (Orco) [15]. After reception of chemical odors, they convert chemical signals into electrical signals that are finally integrated into the central nervous system (CNS) [16]. Orco is more conserved among different species, in contrast to general ORs, and is expressed in almost every ORN [17, 18].

GRs, another kind of chemosensory genes that are expressed in GRNs in taste organs, are more ancient than ORs, but they have same membrane topology as ORs. GRs, which have low sequence identity among insects, also function as ionotropic channels like with ORs [4]. Nevertheless, in *Bombyx mori,* BmorGR1-GR3, identified as carbon dioxide receptors, are conserved [19, 20]*.* Xu and Anderson (2015) expressed HarmGR1, HarmGR2, and HarmGR3 individually in insect Sf9 cells and found that only HarmGR3 could respond to NaHCO_3_ [21]_._ Ning et al. (2016) used two-electrode voltage-clamp recording and concluded that HarmGR1 and HarmGR3 were indispensable and sufficient for sensing CO_2_ through the *Xenopus* oocyte expression system [22]. Meanwhile, gustatory receptors also worked as ‘sugar’ receptors and ‘bitter’ receptors [20, 23].

IRs, evolved from the ionotropic glutamate receptor superfamily (iGluRs), were recently identified to be involved in odorant reception [7, 24]. Generally, IRs mainly detect acids, amines, and other chemicals that cannot be recognized by ORs and are not expressed in ORNs which hold ORs or Orco [7]. Additionally, IRs are also involved in regulating the circadian clock in *D. melanogaster* and are correlated with physical defense in *Daphnia pulex* [25]. In *Drosophila*, IR94b is associated with auditory system functions [26]. However, the function of IRs in Lepidopteran insects remains lesser known.

OBPs and CSPs, belong to a class of small water-soluble proteins containing a hydrophobic pocket, and are impregnated in the sensilla lymph. Generally, insects OBPs share six conserved cysteines while CSPs contain four conserved cysteines [15, 27]. It is believed that OBPs and CSPs play the same role in the chemoreception progress. When the odor molecules enter the sensilla from the surface pores, they can selectively transport odorant molecules to ORNs and benefit the sensitivity of the insect olfactory system [15]. OBPs and CSPs are also found to participate in other physiological processes in addition to chemoreception [28].

SNMPs are expressed in pheromone sensitive ORNs in Lepidoptera and Diptera [29]. There are two types of SNMPs: SNMP1, co-expressed with pheromone receptors; and SNMP2, which are confined to sensilla support cells [29, 30]. SNMP1 has been proven to participate in pheromone signal transduction, but the mechanism of action is unknown.

In the second largest insect order, Lepidoptera, chemosensory genes have been identified by genome and/or transcriptome sequencing in domesticated silkmoth, *B. mori* [31]; the diamondback moth, *Plutella xyllostella* [32, 33]; the cotton bollworm, *Helicoverpa armigera* [34–37]; the rice stem borer, *Chilo suppressalis* [38]; the oriental tobacco budworm, *H. assulta* [36]; and many other moths [39–44]. Identification of these chemosensory genes has laid a solid foundation for further research on the molecular mechanisms of chemosensory reception in these moths.

The oriental armyworm *Mythimna separata* (Walker), an economically important and common Lepidopteran pest, is widely distributed in eastern Asia and Australia, and attacks many crop plants such as maize, sorghum, and rice. The clustering and migratory characteristics of *M. separata* result in widespread incidence and can lead to complete crop loss [45–49]. Compared to chemical pesticides, pheromone-baited trapping is an effective and environmentally friendly method to manage *M. separata*. The sex pheromone of *M. separata* has been identified and is already used to control *M. separata* [50–52]. Unfortunately, pheromone trapping was found to be ineffective for *M. separata* for an unknown reason. Lihuang et al. found that the volatile of *Pterocarya stenoptera* and *Salix babylonica* stimulated electroantennogram (EAG) response of *M. separata* and poplar odors have been used to attract *M. separata* in the field, but, the mechanism of attraction is unknown [53]. To better understand the chemoreception mechanism and to find chemosensory genes in *M. separata*, we assembled and analyzed *M. separata* transcriptomes from three chemosensory organs (antennae, labial palps, and proboscises) using Illumina sequencing technology. In this study, we reported the results including sequencing, gene annotation, and a dataset of 62 ORs, 20 IRs, 16 GRs, 38 OBPs, 26 CSPs, and 2 SNMPs.

## Results

### Transcriptome assembly

The transcriptomes of female antennae, male antennae, labial palps (mix of male and female), and proboscises (mix of male and female) of *M. separata* were separately sequenced using Illumina HiSeq 2000 platform. A total of 100.6 million, 94.1 million, 94.7 million, and 110.9 million raw reads were obtained, respectively. After filtering, 98.5 million, 92.0 million, 92.7 million, and 108.3 million clean reads were generated, respectively. Assemblies led to the generation of 73,342, 71,552, 56,263, and 64,136 unigenes separately for female antenna, male antenna, labial palp, and proboscis. After merging and clustering, a final transcript dataset was revealed, with 71,008 unigenes consisting of 29,388 distinct clusters and 41,620 distinct singletons. The dataset was 72.2 megabases in size and with a mean length of 924 nt and N50 of 1748 nt (Additional file 1: Table S1).

### Identification of candidate odorant receptors

The candidate ORs were identified by key word search of the blastx annotation. Sixty-two putative OR genes were identified in *M. separata*. Of these, 49 unigenes were full-length putative OR genes with complete open reading frames (ORFs) and a general length of 1200 bp and 5–8 transmembrane domains (TMDs), which are characteristic of typical insect ORs.

**Fig. 1:**
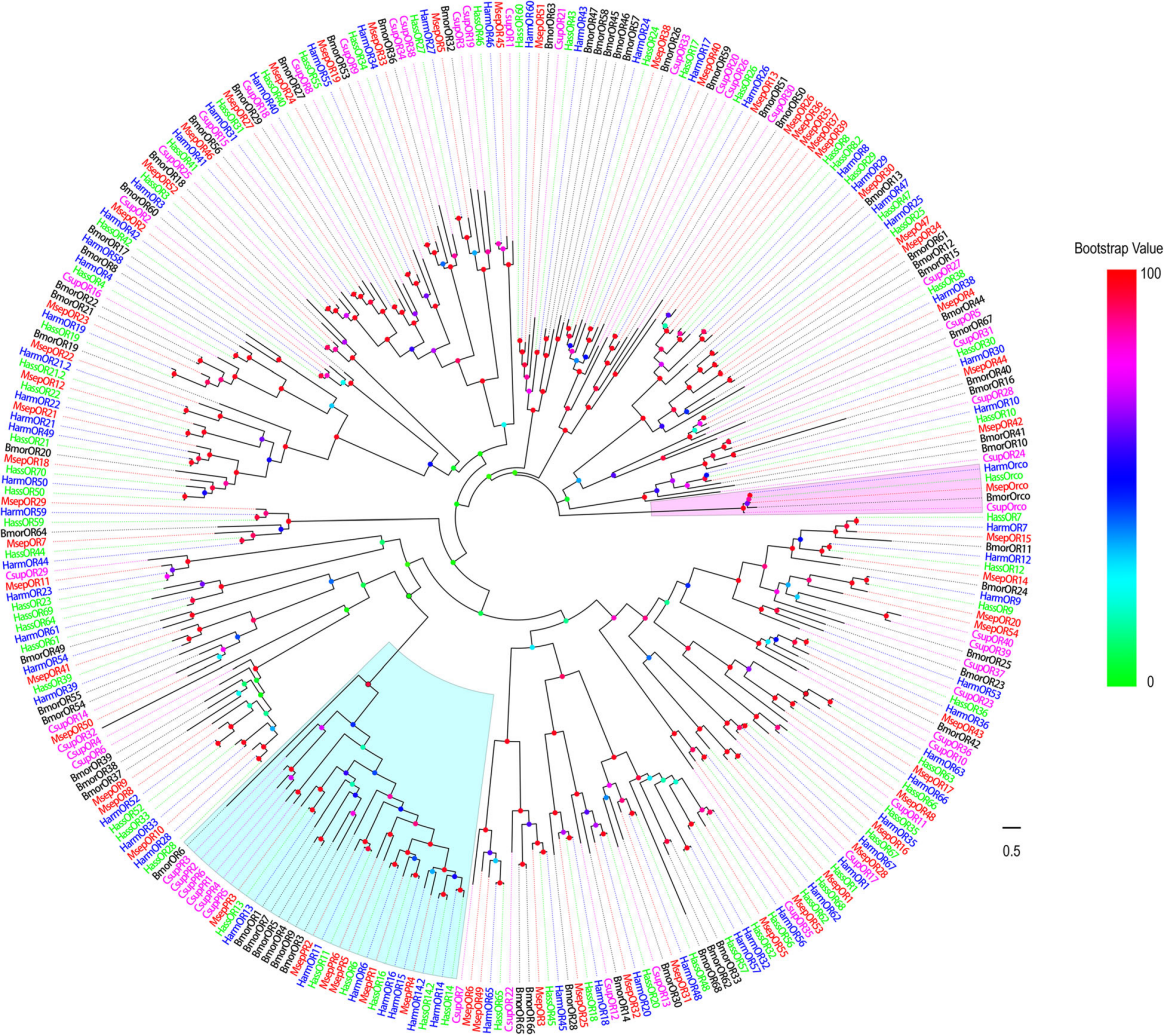
Phylogenetic tree of putative *M. separata* ORs with known Lepidopteran ORs. This tree was constructed using RAxML based on alignment results of MAFFT. Msep: *M. separata* (red); Harm: *H. armigera* (blue); Hass: *H. assulta* (green); Bmor: *B. mori* (black); Csup: *C. suppressalis* (purple). The clade in wine indicates the pheromone receptor gene clade and in purple the Orco co-receptor gene clade

Next, we performed a phylogenetic analysis using our ORs and the ORs from *B. mori* [31], *H. armigera* [34, 36], *C. suppressalis* [38] and *H. assulta* [36] (Fig. 1). The *M. separata* OR co-receptor, named MsepOrco, was easily detected due to its high degree of orthology with the conserved insect co-receptor in other Lepidopteran moths. Six putative pheromone receptors (PRs), named MsepPRx (x = 1 through 6), were easily identified as they shared considerable similarity with other Lepidopteran PRs. Other putative ORs, named MsepOR followed by a numeral in descending order of their coding region lengths, were highly divergent and shared low similarity with other known Lepidopteran ORs. Almost all MsepOR putative proteins were clustered with at least one Lepidopteran orthologous OR in the phylogenetic tree. A species-specific branch was detected including four ORs from *M. separata* (MespOR35, 36, 37 and 39). As expected, the sequence similarity of these four MsepORs was 90.79%. Information including unigene reference, length, best blastx hit, and FPKM (fragments per kb per million fragments) of all 62 putative odorant receptors are listed in Additional file 2: Table S2.

### Identification of candidate ionotropic receptors

The second type of olfactory receptor, IR, belongs to an ancient chemosensory receptor family. In this study, 20 putative IRs were identified in the *M. separata* transcriptome by bioinformatics analysis according to their similarity to known insect IRs. Of these IRs, 13 sequences contained a full-length ORF, the remaining 7 sequences were incomplete due to lacking a 5′ and/or 3′ terminus. Of these, 12 IRs contain three TMDs predicted by TMHMM 2.0 (Additional file 2: Table S2), which was consistent with the characteristics of insect IRs.

**Fig. 2:**
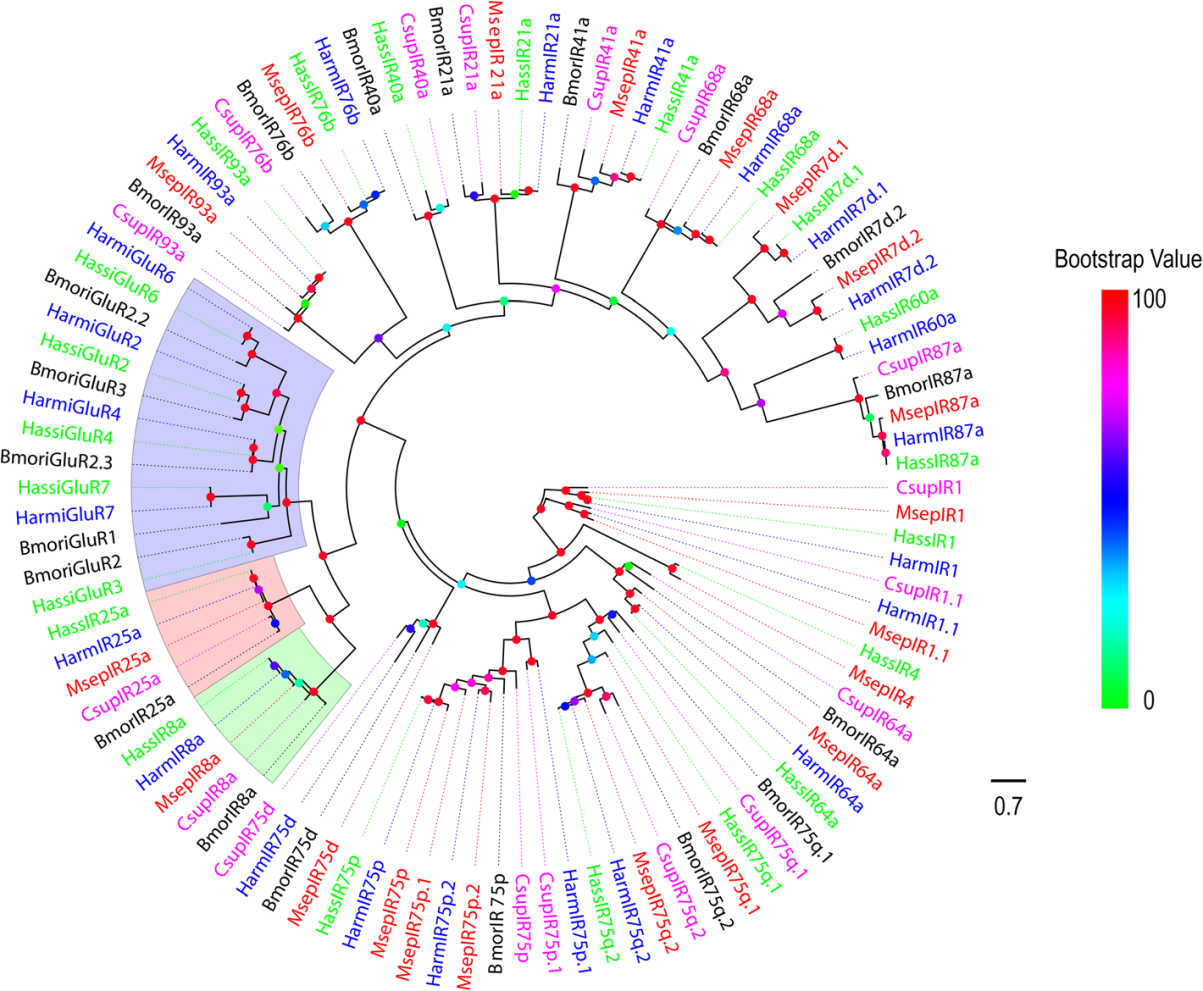
Phylogenetic tree of putative *M. separata* IRs with known Lepidopteran IRs. This tree was constructed using RAxML based on alignment results of MAFFT. Msep: *M. separata* (red); Harm: *H. armigera* (blue); Hass: *H. assulta* (green); Bmor: *B. mori* (black); Csup: *C. suppressalis* (purple). The clade in purple indicates the ionotropic glutamate receptor gene clade, in wine the ionotropic receptor 25a clade, and in green the ionotropic receptor 8a clade

To distinguish putative IRs from ionotropic glutamate receptors (iGluRs), a phylogenetic analysis was conducted using putative *M. separata* IRs with iGluRs of *H. armigera*, *H. assulta*, *B. mori* iGluRs, and with IRs of *B. mori* [54], *H. armigera* [34, 36], *C. suppressalis* [38], *H. assulta* [36]. A clear segregation between iGluRs and IRs were revealed in the phylogenetic tree. The *M. separata* candidate IRs were clustered with other known Lepidopteran IRs into a separate clade (Fig. 2). According to their positions in the phylogenetic tree and based on strong bootstrap support, all candidate IRs were given names consistent with the number and suffix of the other known Lepidopteran IR orthologues in the same clade. The information including unigene reference, length, best blastx hit, and FPKM of all the IRs are listed in Additional file 2: Table S2.

### Identification of candidate gustatory receptors

Sixteen putative GRs were identified in the *M. separata* transcriptome. Four GR sequences contained a full-length ORF; the remaining 12 sequences were incomplete due to lacking a 5′ and/or 3′ terminus (Additional file 2: Table S2).

**Fig. 3:**
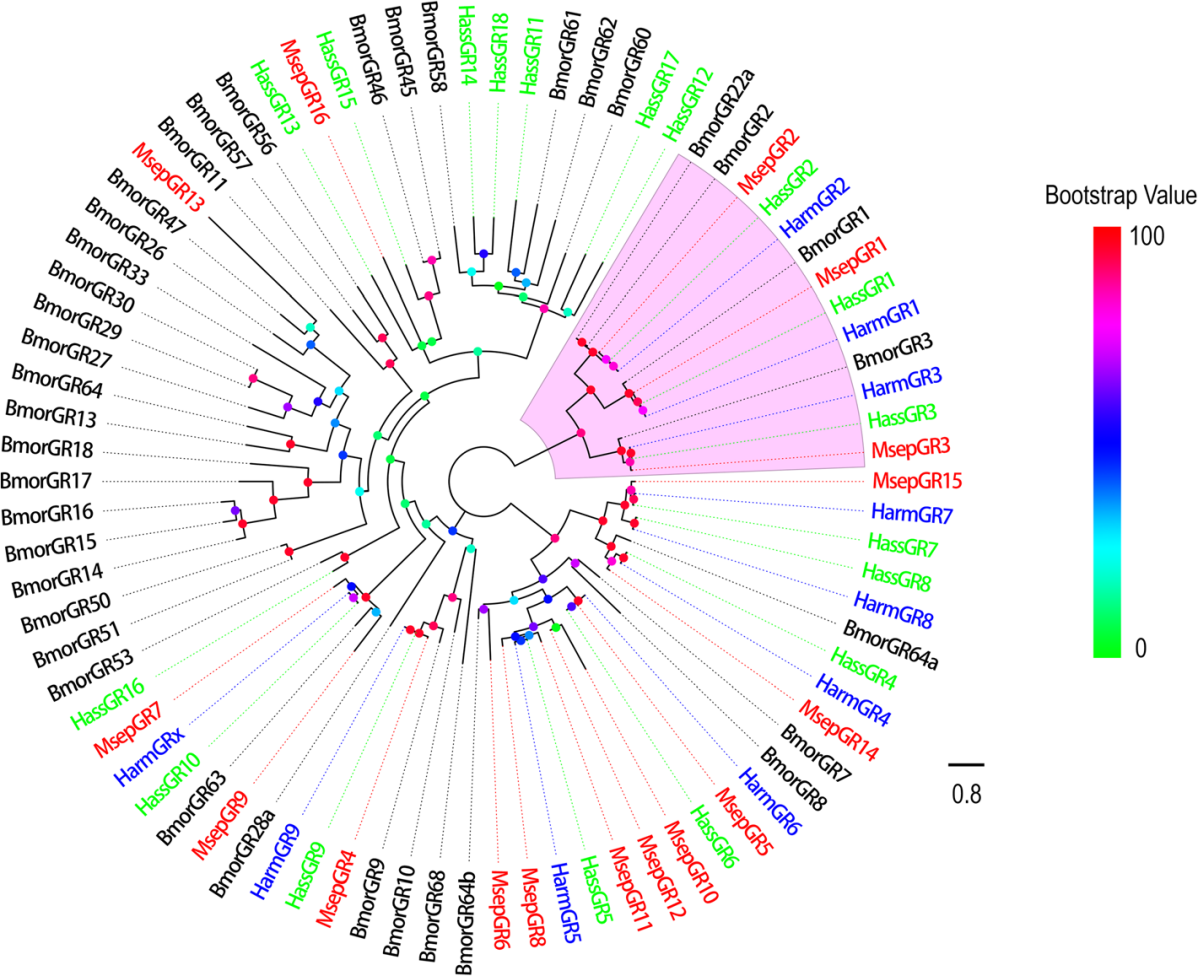
Phylogenetic tree of putative *M. separata* GRs with known Lepidopteran GRs. This tree was constructed using RAxML based on alignment results of MAFFT. Msep: *M. separata* (red); Harm: *H. armigera* (blue); Hass: *H. assulta* (green); Bmor: *B. mori* (black). The clade in purple indicates the carbon dioxide receptors group

To identify GRs in *M. separata*, the putative proteins were phylogenetically analyzed with known Lepidopteran moth GRs including CO_2_ receptors from *B. mori* [19, 20], *H. armigera* [35], and *H. assulta* [55]. Three GRs of *M. separata*, named MsepGR1, MsepGR2, and MsepGR3, were clustered into CO_2_ GRs clade with BmorGR1, 2, 3; HarmGR1, 2, 3; and HassGR1, 2, 3 (Fig. 3). Information including unigene reference, length, best blastx hit, and FPKM of all putative GRs are listed in Additional file 2: Table S2.

### Identification of putative odorant binding proteins

Thirty-eight putative unigenes encoding OBPs, including 2 general odorant binding proteins (GOBPs) and 3 pheromone binding proteins (PBPs), were identified from *M. separata* transcriptome by bioinformatics analysis. Among them, 33 were full-length putative OBP genes and the remaining 5 sequences were incomplete due to lacking a 5′ or 3′ terminus (Additional file 2: Table S2).

**Fig. 4:**
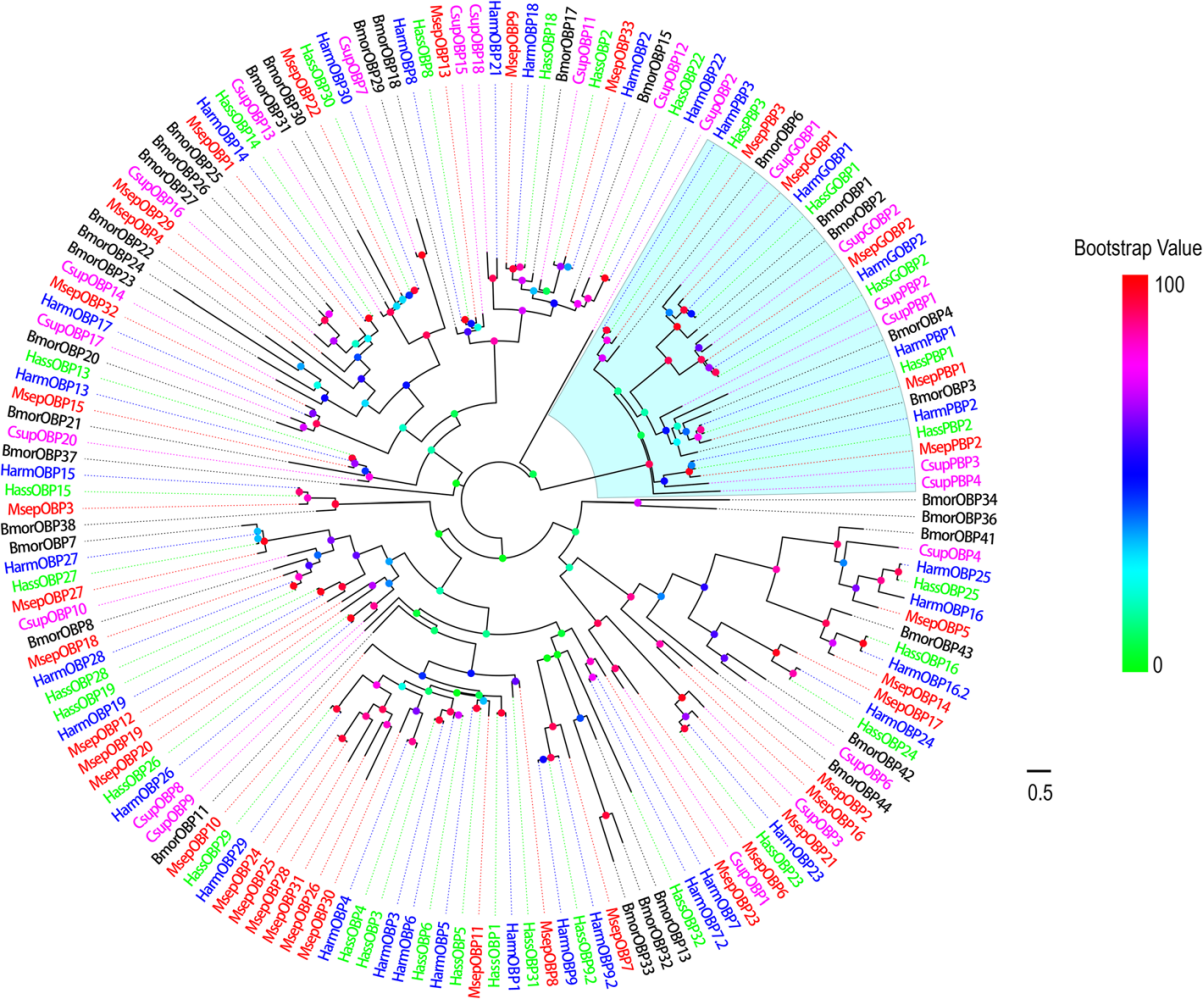
Phylogenetic tree of putative *M. separata* OBPs with known Lepidopteran OBPs. This tree was constructed using RAxML based on alignment results of MAFFT. Msep: *M. separata* (red); Harm: *H. armigera* (blue); Hass: *H. assulta* (green); Bmor: *B. mori* (black); Csup: *C. suppressalis* (purple). The clade in purple indicates general odorant binding proteins and pheromone binding proteins

Phylogenetic analysis was performed with OBPs containing PBPs and GOBPs from *B. mori* [56], *H. armigera* [34, 36], *C. suppressalis* [38], and *H. assulta* [36]. In the phylogenetic tree, the PBPs and GOBPs sequences were clustered into the PBP and GOBP clades, respectively (Fig. 4). MsepOBP24 and MsepOBP25 shared 97.78% sequence identity and clustered together. However, MsepOBP19 and MsepOBP20 and Mesp26 and MsepOBP31 only shared 17.69 and 14.84% similarity, respectively; they were also clustered together. All candidate OBPproteins were clustered with at least one Lepidopteran orthologue. A special group of MsepOBPs containing 5 members (MsepOBP24, 25, 26, 28 and 31) was found. They formed a clade with HarmOBP29 and HassOBP29. The *M. separata* GOBPs were named MsepGOBP1 and MsepGOBP2 following NCBI records. The PBPs and remaining OBPs were named MsepPBP and MsepOBP followed by a number in descending order of their coding region lengths. Information including unigene reference, length, best blastx hit and FPKM of all putative 38 unigenes are listed in Additional file 2: Table S2.

### Identification of putative chemosensory proteins

In this study, 26 putative unigenes encoding CSPs were identified. Among these unigenes, 21 sequences were full-length putative CSP genes because they had complete ORFs and 4 cysteines, which are characteristic of typical insect CSPs. The remaining 5 sequences were incomplete due to lacking a 5′ or 3′ terminus (Additional file 2: Table S2).

**Fig. 5:**
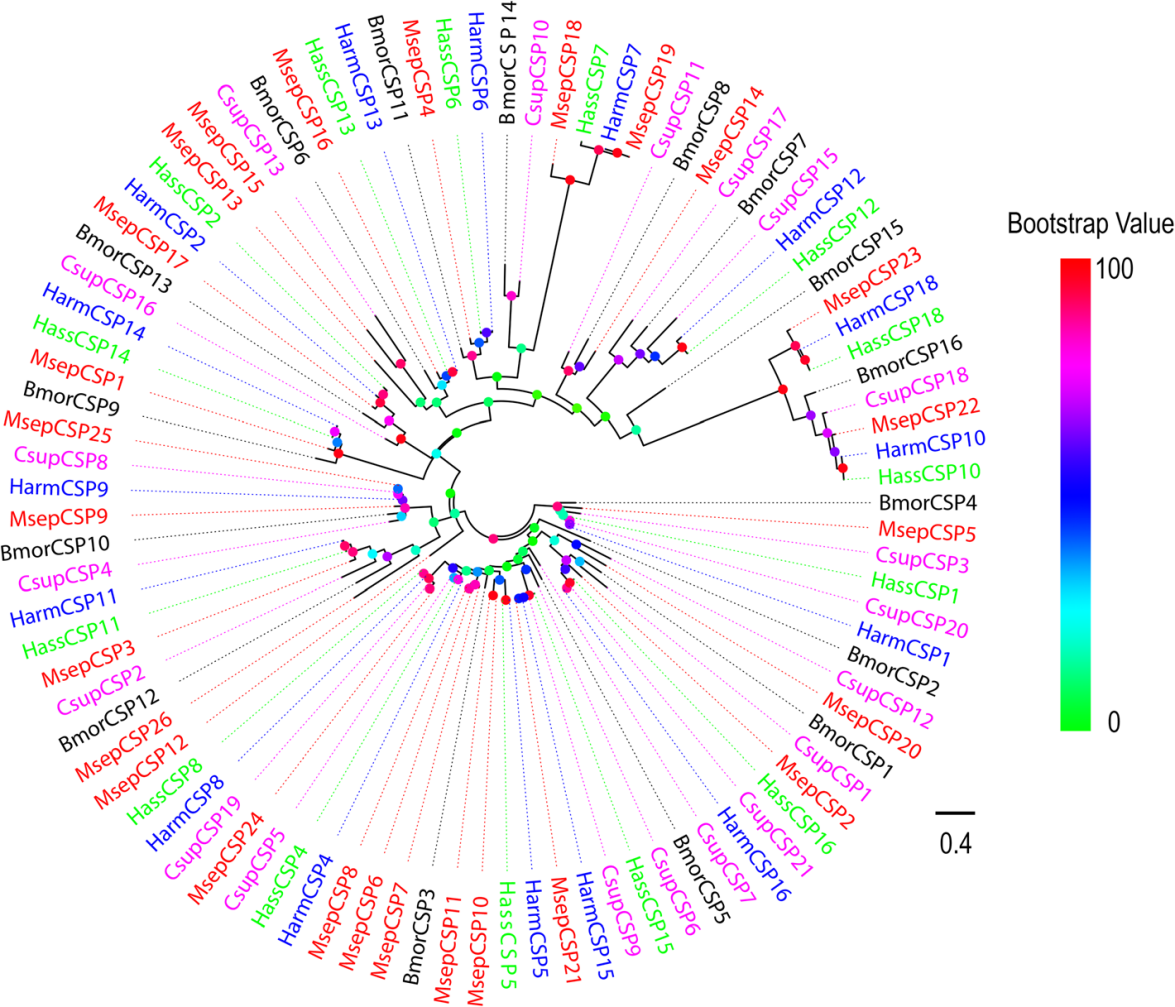
Phylogenetic tree of putative *M. separata* CSPs with known Lepidopteran CSPs. This tree was constructed using RAxML based on alignment results of MAFFT. Msep: *M. separata* (red); Harm: *H. armigera* (blue); Hass: *H. assulta* (green); Bmor: *B. mori* (black); Csup: *C. suppressalis* (purple)

Phylogenetic analysis was performed with CSPs from *B. mori* [57], *H. armigera* [34, 36], *C. suppressalis* [38], and *H. assulta* [36]. In the phylogenetic tree, all candidate CSP proteins were clustered with at least one Lepidopteran orthologue (Fig. 5). The *M. separata* CSPs were named MsepCSP followed by a number in descending order of their coding region lengths. Information including unigene reference, length, best blastx hit and FPKM of all the 26 CSPs are listed in Additional file 2: Table S2.

### Identification of candidate sensory neuron membrane proteins

Two unigenes encoding SNMPs, named MsepSNMP1 and MsepSNMP2 were identified in *M. separata* transcriptome by bioinformatics analysis. These two unigenes were full-length SNMP genes as they had complete ORFs with lengths of more than 2000 bp. Information including unigene reference, length, best blastx hit, and FPKM of two SNMPs are listed in Additional file 2: Table S2.

### Differentially expressed genes (DEGs) analysis

**Fig. 6:**
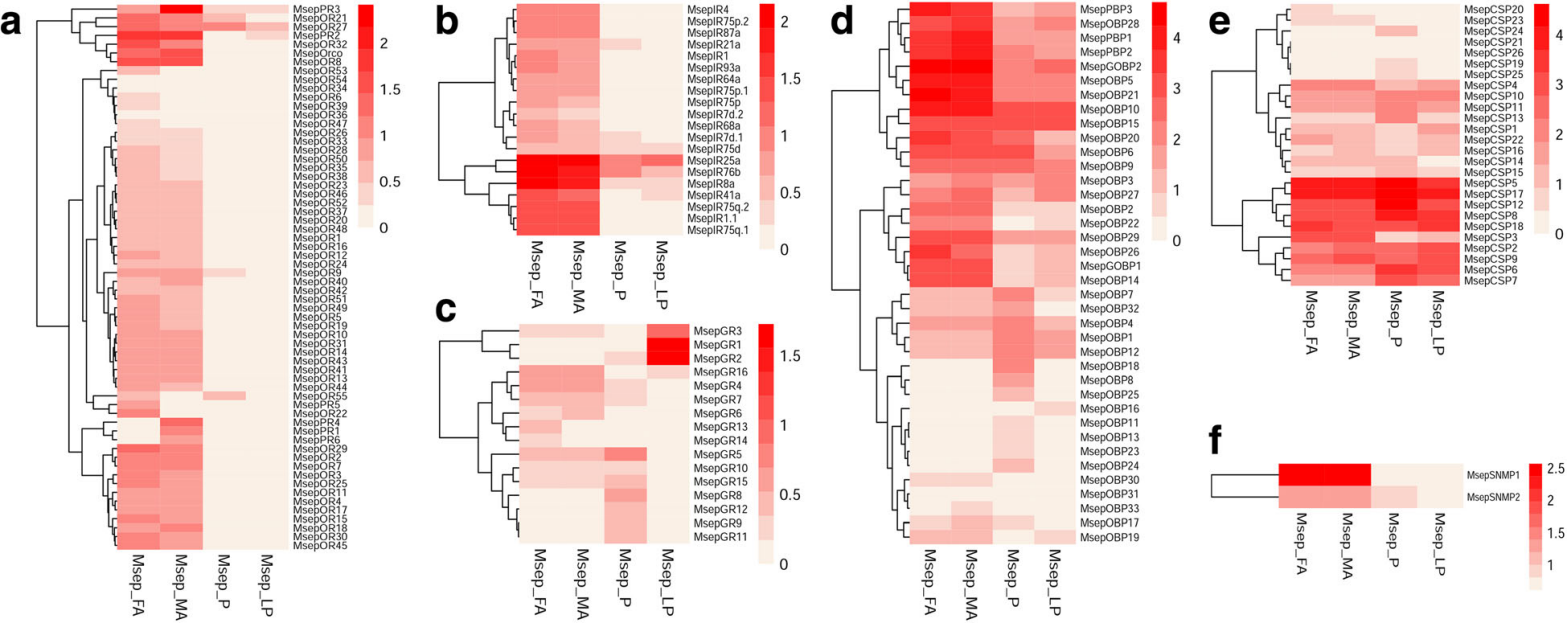
Expression profiles of chemosensory genes in *M. separata*. A: ORs; B: IRs; C: GRs; D: OBPs; E: CSPs; F: SNMPs. FA: female antennae; MA: male antennae; P: proboscis; LP: labial palp

Gene expression levels of all candidate chemosensory genes in male and female antennae, proboscises, and labial palps were assessed by analyzed differentially expressed genes (DEGs) using fragments per kilobase per million fragments (FPKM) values, represented in a heatmap (Fig. 6). All candidate ORs were highly expressed in antennae. Most of the candidate ORs showed antennal-specific or antennal-biased expression pattern. Only four candidate ORs (MsepOR9, 21, 27, and 55) showed relatively high expression levels in proboscises. Relatively high expression of MsepOR27 was also detected in labial palps. MsepIRs showed a similar expression profile to MsepORs, but more IRs could be detected in proboscises and labial palps. MsepGRs were mainly expressed in proboscises and labial palps and MsepGR1and MsepGR2 had the highest expression level in labial palps. MsepOBPs and MsepCSPs both exhibited diverse expression patterns. MsepSNMP1 was highlyexpressed in antennae, however MsepSNMP2 were widely expressed in all the test tissues.

### Tissue- and sex- specific expression of candidate *M. separata* OR, IR, and GR genes

To confirm expression profiles of the three families of receptor genes, real-time quantitative RT-PCR (qPCR) was performed using the eight different samples including female antennae, male antennae, female proboscises, male proboscises, female labial palps, male labial palps, legs (mix of female and male), and thoraxes and abdomens (mix of female and male).

**Fig. 7:**
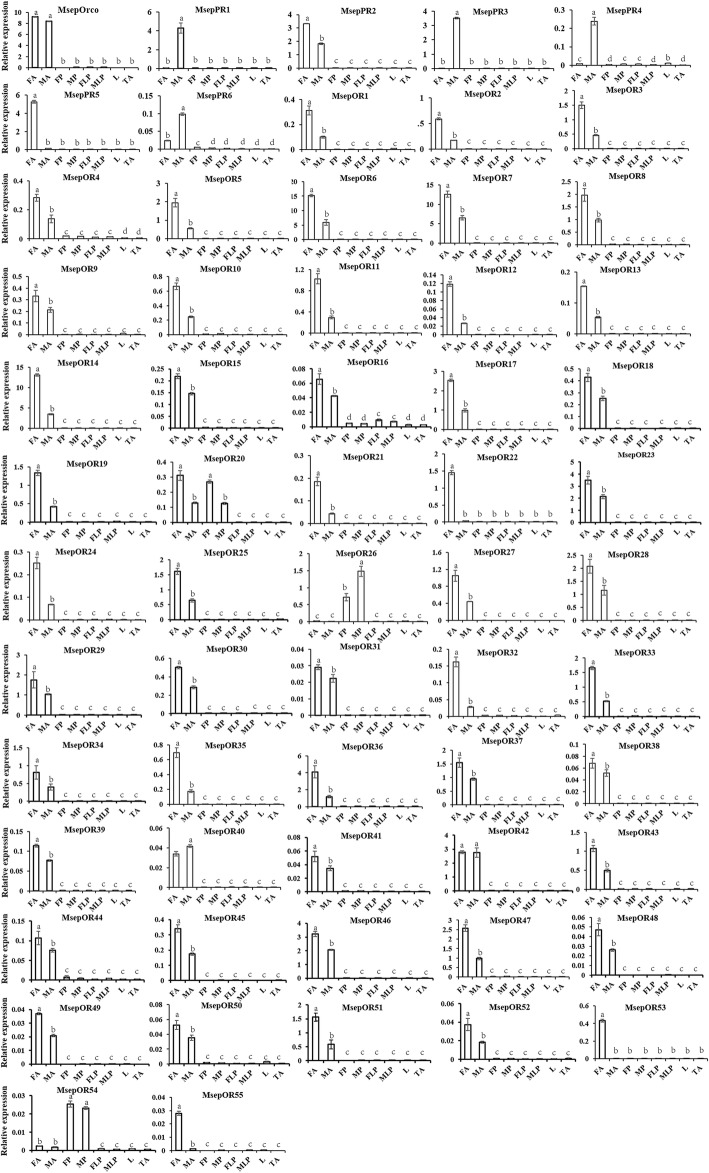
Tissue- and sex- specific expression of *M. separata* candidate OR genes. FA: female antennae; MA: male antennae; FP: female proboscis; MP: male proboscis; FLP: female labial palp; MLP: male labial palp; L: legs (both sexes mixed); TA: thorax and abdomen (both sexes and tissues mixed). Y-axis is relative expression to reference gene *MsepRPS3* (2^-∆CT^) (mean + standard error). Bars labeled with different letters are significantly different (*p* < 0.05, ANOVA, LSD)

Expression levels of all 62 candidate ORs were successfully detected in qPCR analysis (Fig. 7). These results indicated that all candidate ORs were antennae enriched, except for MsepOR26 and MespOR54, which were highly expressed in proboscises. MsepOrco was equally expressed in the antennae of both sexes. Of 6 candidate PRs, MsepPR1, MsepPR3, and MsepPR4 were expressed specifically in the male antennae and MsepPR6 had a higher expression level in male antennae than in female antennae. In contrast, MsepPR2 had higher expression in female antennae than in male antennae. MsepPR5 was expressed specifically in the female antennae. Of the remaining candidate ORs, most candidate ORs had higher expression in female antennae than in male antennae.

**Fig. 8:**
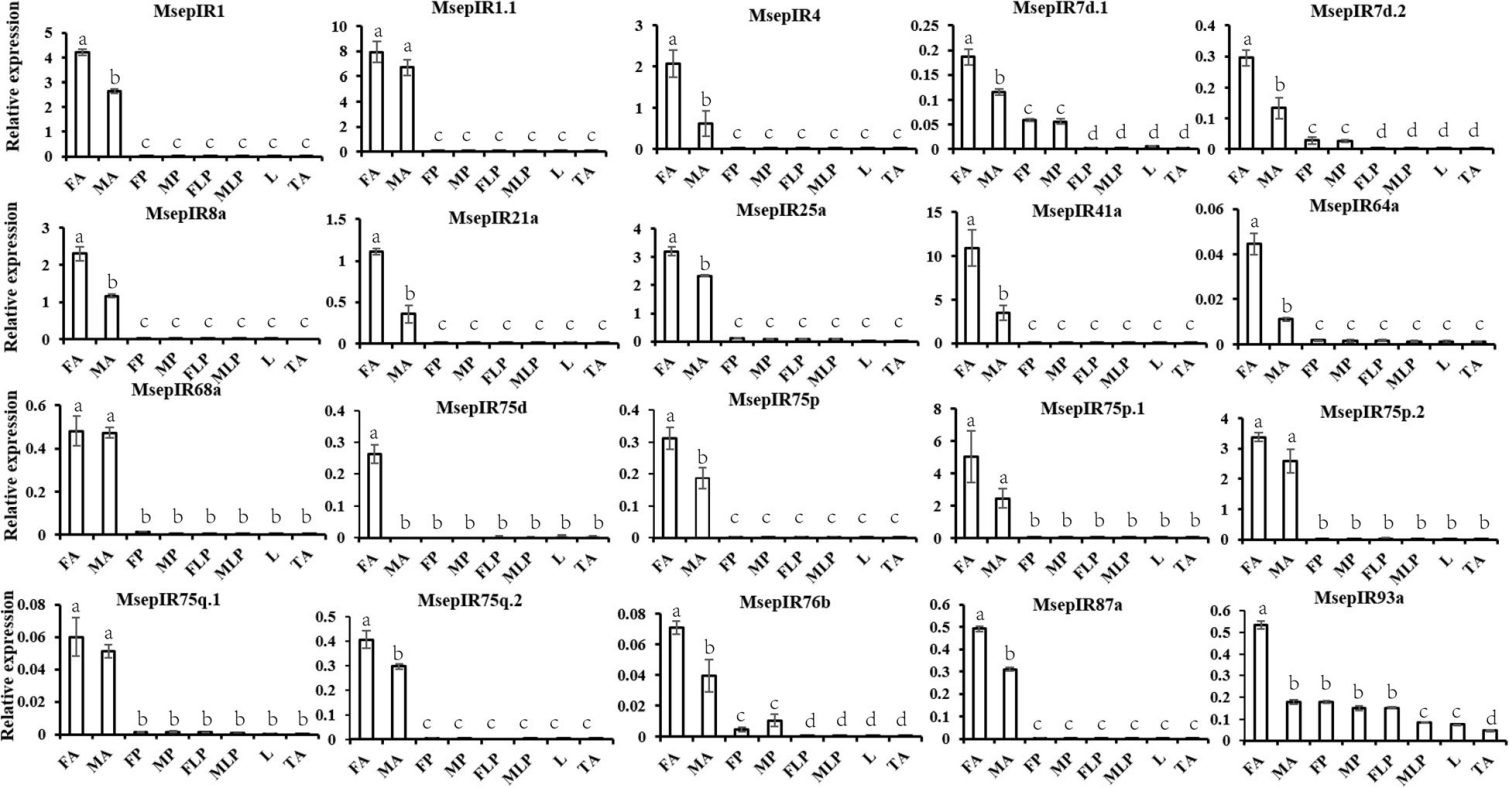
Tissue- and sex- specific expression of *M. separata* candidate IR genes. FA: female antennae; MA: male antennae; FP: female proboscis; MP: male proboscis; FLP: female labial palp; MLP: male labial palp; L: legs (both sexes mixed); TA: thorax and abdomen (both sexes and tissues mixed). Y-axis is relative expression to reference gene *MsepRPS3* (2^-∆CT^) (mean + standard error). Bars labeled with different letters are significantly different (*p* < 0.05, ANOVA, LSD)

Expression of all 20 candidate IRs was successfully detected in qPCR analysis (Fig. 8). These results indicated that most of the candidate IRs were expressed in the olfactory organ antennae, and not in the non-olfactory organs such as legs, thoraxes and abdomens, except MsepIR93a, which was widely expressed in olfactory and non-olfactory organs. Among them, MsepIR68a, MsepIR75p.2, and Msep75q.1 had the same expression level between male and female antennae and the other IRs had higher expression in female antennae than male antennae sharing the similar trend of the FPKM values (Additional file 2: Table S2).

**Fig. 9:**
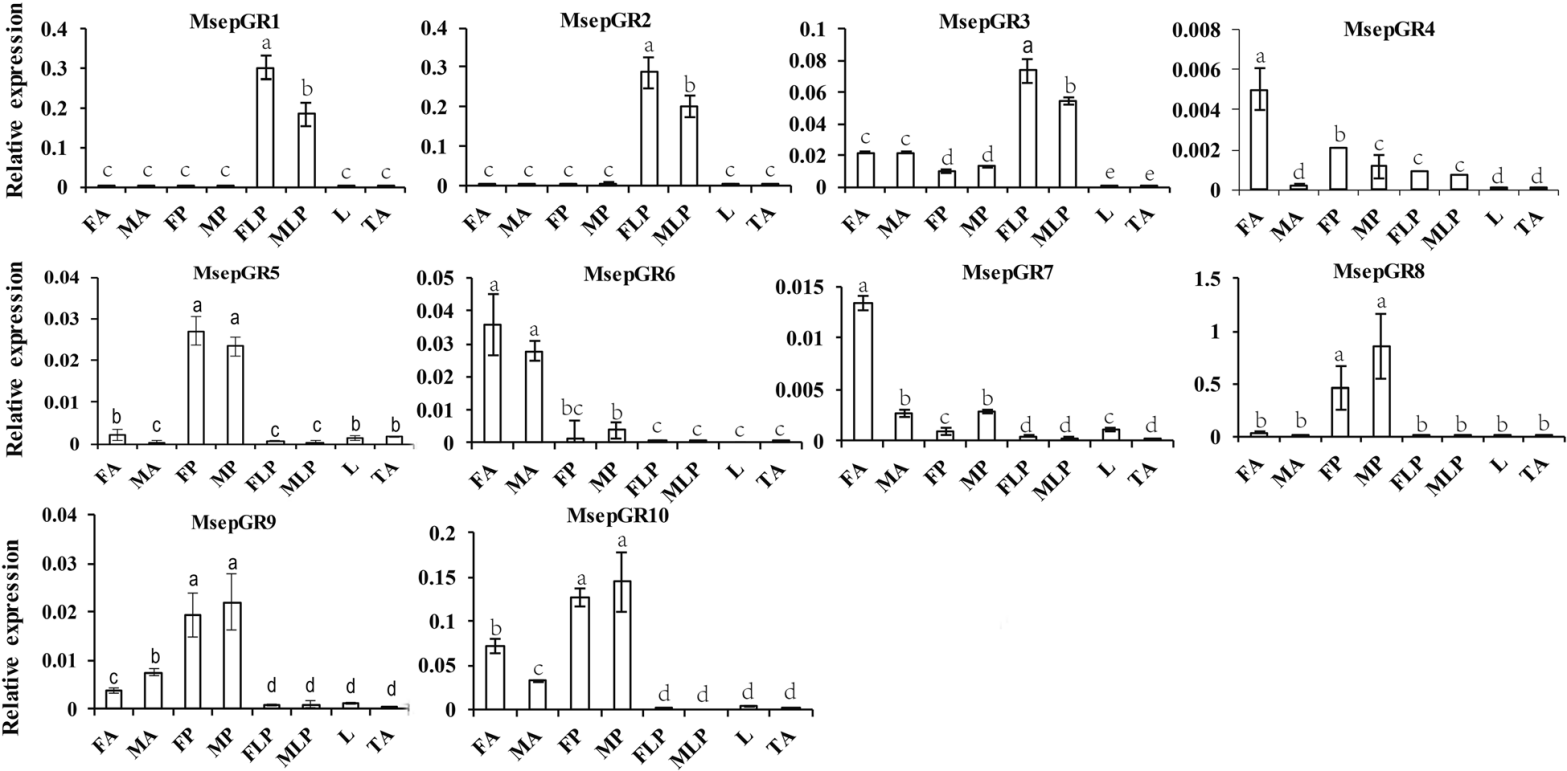
Tissue- and sex- specific expression of *M. separata* candidate GR genes. MsepGR1, MsepGR2 and MsepGR3, expressed in labial pales, were identified as candidate CO_2_ receptors. FA: female antennae; MA: male antennae; FP: female proboscis; MP: male proboscis; FLP: female labial palp; MLP: male labial palp; L: legs (both sexes mixed); TA: thorax and abdomen (both sexes and tissues mixed). Y-axis is relative expression to reference gene *MsepRPS3* (2^-∆CT^) (mean + standard error). Bars labeled with different letters are significantly different (*p* < 0.05, ANOVA, LSD)

Expression levels of the 10 candidate GRs were detected in qPCR analysis (Fig. 9). MsepGR1 and MsepGR2 were selectively expressed in labial palps and had higher expression in female labial palps. MsepGR3 had the highest expression level in female labial palps compared to other organs. There were 4 proboscis-enriched GRs (MsepGR5, MsepGR8, MsepGR9 and MsepGR10), all of which showed no significant difference between male and female proboscises. MsepGR4 and MsepGR7 shared the same expression profile and both had the highest expression in female antennae. MsepGR6 had a higher expression level in antennae and there was no difference between male and female antennae. Most candidate GRs detected were expressed in different tissues with the similar trend to the FPKM values (Additional file 2: Table S2).

## Discussion

A dataset of candidate ORs (62), IRs (20), GRs (16), OBPs (38), CSPs (26), and SNMPs (2) were identified in *M. separata* using transcriptome sequencing. Three ORs (GenBank accessions BAG71423.2, BAG71415.1 and BAG71414.1), four IRs (GenBank accessions ARB05665.1, ARB05666.1, ARB05667.1, and ARB05668.1) and 1 OBP (GenBank accession BAG71416.1) previously annotated in *M. separata* available in NCBI were identified in our dataset. There are three previous studies antennal on transcriptome of *M. separata.* He et al. (2017) identified 126 olfactory genes including 43 ORs, 13 GRs, 16 IR, 37 OBPs, 14 CSPs, and 3 SNMPs [58], Chang et al. (2017) obtained 130 chemosensory genes encoding 71 ORs, 1 GR, 8 IRs, 32 OBPs, 16 CSPs, and 2 SNMPs [59] and Liu et al. (2017) gained 60 ORs, 8 GRs, 24 IRs, 50 OBPs, 22 CSPs and 2 SNMPs [60]. Compared with Liu’s study which was also on the head transcriptome of *M. separate*, we identified some novel chemosensory genes and the lengths of the genes were significantly longer. In this study, we obtained 64 novel chemosensory genes including 27 ORs, 11 GRs, 9 IRs, 7 OBP, and 10 CSPs compared with Liu’s study. And the lengths of the genes we identified were significantly longer. We even found the some transcripts reported in previous study were from one chemosensory gene identified in this study. This is due to the much deeper sequencing we performed compared to previous studies. We also found some chemosensory genes Liu identified were missing in our study including 10 ORs, 2 GRs, 2 IRs, 16 OBP, and 5 CSPs (Additional file 3). This is because Liu et al. sequenced the transcriptomes from the heads of the larvae, pupae and adults. We just sequenced the transcriptomes antennae, labial palps, and proboscises of adults, so the chemosensory genes expressed in other tissue on head of adults and other development stages were impossible to be detected. Moreover, because we sequenced the transcriptomes of male antennae, female antennae, proboscises and labial palps separately, we were able analyzed the expression levels of chemosensory genes in these four samples. Our results provide a foundation for identify the mechanism of chemical communication in *M. separata*.

A total of 62 ORs were identified in the *M. separata* transcriptome. Our dataset of 62 ORs were similar in quantity to the antennal transcriptomes of *H. armigera* with 66 ORs [34, 36], *H. assulta* with 64 ORs [36], *C. suppressalis* with 47 ORs [38], and *B. mori* with 62 ORs [31]. Six candidate PRs were identified by their similarities to other known Lepidopteran PRs and physiological analysis. The number of PRs is consistent with the classic number of PRs in noctuidae moths first identified in *Heliothis virescens* [61] and then in other species. In recent studies, more than 6 PRs have been identified in *H. armigera* [34, 36], *P. xylostella* [33], and other species. Five of the 6 candidate PRs showed a male-specific or male-biased expression profile. Interestingly, MsepPR5 was specifically expressed in the female antennae. In the previous study in *P. xylostella*, a female specific PR was identified by transcriptome sequencing [33]. The phenomenon suggested that female-specific PRs are not rare in moths. These PRs cluster with pheromone receptors and have a female-specific expression profile. Such special PRs may respond to some male specific chemicals that have similar structures to female sex pheromones. Almost all MsepORs showed female antennae highly expressed except for MsepOR26 and MsepOR54. Both of them were highly expressed in proboscises indicating that they might participate in feeding.

IRs, another class of chemosensory receptors, were first identified in *D. melanogaster* genome [8]. In our study, 20 candidate IRs were identified in the *M. separata* transcriptome including two co-receptors, MsepIR8a and MsepIR25a. Compared to ORs, the IR family is relatively conserved both in sequence and expression pattern. In the expression levels of 20 MsepIRs we identified, MsepIR75d was specifically expressed in female antennae, and MsepIR4, MsepIR21a, MsepIR41a, MsepIR64a, and MsepIR75q.1 showed significantly different expression between male and female antennae. The functions of IRs, which have been mainly studied in *D. melanogaster*, include sensing odor, taste, and temperature, so the functions of these specific expressed MsepIRs need to be further investigated. Interestingly, IR60a, the conserved antennal orthologue, was not found in our results. MespIR40a was also lacking from our transcriptome assemblies. Considering the relatively high sequence conservation, the functions of MsepIRs are probably conserved as IRs in other Lepidopteran moths.

We identified 16 putative GRs in the *M. separata* transcriptome, including 3 GRs for carbon dioxide (MsepGR1, MsepGR2, and MsepGR3) expressed in the labial palps. The results for CO_2_ receptors identified in *M. separata* were similar to the CO_2_ receptors in *Drosophila* [19, 20] and *H. armigera* [21], according to phylogenetic and expression analyses. MsepGR4 and MsepGR7 were expressed much more highly in female antennae, suggesting that they might associated with the process of feeding on nectar in female.

## Conclusions

Our goal for this study was to identify chemosensory genes important for chemoreception in *M. separata*. Our study provided a dataset of candidate 62 ORs, 20 IRs, 16 GRs, 38 OBPs, 26 CSPs, and 2 SNMPs identified in the *M. separata* transcriptome using the Illumina HiSeq 2000 platform. This study provides valuable information for further functional studies of the chemosensory system of *M. separata* at the molecular level, and for further studies of chemoreception mechanisms in Lepidopteran moths.

## Methods

### Insects rearing and tissues collection

The larvae of *M. separata* were collected in Xinxiang, Henan Province, China and the colony was maintained at the laboratory of Henan Agricultural University, Zhengzhou, China. Larvae were reared on an artificial diet and conditions were keep constant at 28 ± 1 °C, 70% ± 5% relative humidity, and a 14 h: 10 h light: dark (L: D) photoperiod. Pupae of different sexes were kept separately in glass tubes (Φ = 2.0 cm, height = 8 cm) until eclosion. Adult male and female moths were fed with 10% sugar solution. Antennae, proboscises, and labial palps of unmated male or female individuals were collected 3–4 days after eclosion, immediately frozen in liquid nitrogen, and stored at − 70 °C for RNA extraction.

### RNA extraction

Total RNA of antennae, proboscises and labial palps were extracted separately from approximately 200 adult male or female moths using TRIzol reagent (Invitrogen, Carlsbad, CA, USA) following the manufacturer’s instruction. Total RNA was dissolved in RNase-free water and RNA integrity was verified by gel electrophoresis. RNA concentration and purity were measured on a Nanodrop ND-2000 spectrophotometer (NanoDrop products, Wilmington, DE, USA).

### cDNA library construction and sequencing

Three micrograms of total RNA from antennae, proboscises, and labial palp of male and female moths (mixed by equal amount) were used to construct four cDNA libraries separately. The libraries were sequenced using the PE100 strategy on the Illumina HiSeqTM 2000 platform (Illumina, San Diego, CA, USA) and performed at the Beijing Genome Institute (Shenzhen, China) following the detailed protocol described in previous studies.

### Assembly

Datasets of clean reads were generated from the raw-reads through the following procedure: 1) reads with adaptors or containing unknown nucleotides at more than 10% were removed directly; 2) low-quality reads containing more than 40% suspect nucleotides with a Phred Quality Score less than 20 were filtered out; and 3) both ends of reads were evaluated to trim unreliable ends containing more than 3 successive suspect nucleotides. All clean-read datasets of female antenna, male antenna, proboscis, and labial palp mixed were fed to Trinity (version 20,120,608) for de novo transcriptome assembly using the paired reads mode with default parameters. [62]. Then the Trinity outputs were clustered by TGICL [63]. The consensus cluster sequences and singletons make up the final unigenes dataset.

### Identification of chemosensory genes

Unigenes were annotated using blastx against the NCBI non-redundant (nr) sequences with e-value <1e-5. Candidate unigenes encoding putative ORs, IRs, OBPs, CSPs, SNMPs, and GRs were selected according to the nr annotation result in the remote sever. All candidate chemosensory genes were manually checked using the blastx program against the nr database. The open reading frames (ORFs) of all putative chemosensory proteins were predicted using the ExPASy (the Expert Protein Analysis System) server (http://web.expasy.org/translate/) [64]. The transmembrane domains (TMDs) of ORs, IRs, and GRs were predicted using TMHMM server version 2.0 (http://www.cbs.dtu.dk/services/TMHMM/) [65]. Putative N-terminal signal peptides of OBPs and CSPs were predicted using SignalP 4.0 server version (http://www.cbs.dtu.dk/services/SignalP/) with default parameters [66].

### Phylogenetic analysis

Alignments of amino acid sequences were performed by MAFFT (https://www.ebi.ac.uk/Tools/msa/mafft/). Phylogenetic trees of chemosensory genes from *M. separata* and other moths were constructed using RAxML version 8 with the Jones-Taylor-Thornton amino acid substitution model (JTT) same as previous research [67]. Node support was assessed using a bootstrap method based on 1000 replicates. The OR data set contained OR sequences identified in four other Lepidoptera moths (65 from *H. assulta* [36], 65 from *H. armigera* [34, 36] 47 from *C. suppressalis* [38] and 62 from *B. mori* [31]). The GR data set contained GR sequences identified in three other Lepidoptera moths (18 from *H. assult* [55], 10 from *H. armigera* [35] and 38 from *B. mori* [20, 23]). The IR data set contained IR sequences identified in four other Lepidopteran moths (17 from *H. assulta* [36], 19 from *H. armigera* [34, 36], 17 from *C. suppressalis* [38]and 15 from *B. mori* [54]). The OBP data set contained OBP sequences identified in four other Lepidopteran moths (30 from *H. assulta* [36], 34 from *H. armigera* [34, 36], 24 from *C. suppressalis* [38] and 33 from *B. mori* [56]). The CSP data set contained CSP sequences identified in four other Lepidopteran moths (15 from *H. assulta* [36], 16 from *H. armigera* [34, 36], 20 from *C. suppressalis* [38] and 16 from *B. mori* [57]).

### DEG analysis

To compare expression levels of chemosensory genes among olfactory organs from males and females, map-based expression profiling analysis was conducted. SOAPaligner (http://soap.genomics.org.cn /soapaligner.html) was applied to remap all clean reads onto the transcript following the principle of up to three base pair mismatches and a minimum length of 40 bp. Transcription levels of all chemosensory genes were reported in values of Fragments Per Kilobase of transcript per million mapped reads (FPKM), the most commonly used method for comparing gene expression levels [12, 68–70]. The hierarchical clustering was generated using Spearman correlation coefficients of log2-transformed FPKM expression values. Heat maps of differential gene expression in male and female antennae, proboscises, and labial palps were generated using R pheatmap packages. [71].

### Tissue- and sex- specific expression analysis by real-time quantitative PCR

Real-time quantitative PCR (qPCR) was performed to verify the expression of candidate ORs, IRs, and MsepGR1–10. The six GRs were not analyzed because of their short length. Male and female antennae, proboscises, labial palps, legs, thoraxes, and abdomens were collected from 3-day old adult *M. separata* after eclosion and total RNA was extracted using TRIzol reagent (Invitrogen, Carlsbad, CA, USA). The cDNA was synthesized from total RNA using RevertAid First Strand cDNA Synthesis Kit (Thermo Scientific, Waltham, MA, USA). Gene specific primers were designed using Primer 5 (Additional file 4: Table S3) and synthesized by Sangon Biotech Co., Ltd. (Shanghai, China). A total of eight samples including female antennae, male antennae, female proboscises, male proboscises, female labial palps, male labial palps, legs (male and female mixture), and thoraxes & abdomens (male and female mixture) were subjected to qPCR to verify tissue-specific expression and sex-specific expression of candidate chemosensory receptor genes. qPCR was carried out using the GoTaq qPCR Master Mix (Promega, Madison, WI, USA) on an Applied Biosystems 7500 Fast Real-Time PCR System (ABI, Carlsbad, CA, USA). The reaction conditions were set as follows: 95 °C for 2 min; 40 cycles of 95 °C for 15 s, and 60 °C for 50 s. Each qPCR reaction for every sample was performed three times to check reproducibility. And the melt curves were gotten attention to check the specificity of Primers. The candidate genes’ relative expression was quantified using the comparative 2^-∆CT^ method [72]. Chemosensory receptors’ expression levels were calculated relative to the reference gene *MsepRPS3.* Data analysis was conducted using SPSS 16.0 (SPSS Inc., Chicago, IL, USA). The significant difference analysis of target genes among various organs was determined using ANOVA (one-way nested analysis of variance), following by LSD (Least-Significant Difference) tests.

## Additional files

Additional file 1 **Table S1**. Assembly summary of *M. separata* antennal transcriptome. (DOCX 18 kb)

Additional file 2 Table S2. Candidate chemosensory genes of *M. separata* antennal transcriptome. Table S2–1: Unigenes of candidate odorant receptors with gene name, length, ORF, best blastx hit and identity, etc. Table S2–2: Unigenes of candidate ionotropic receptors. Table S2–3: Unigenes of candidate gustatory receptors. Table S2–4: Unigenes of candidate odorant binding proteins. Table S2–5: Unigenes of candidate chemosensory proteins. Table S2–6: Unigenes of candidate sensory neuron membrane proteins. (DOCX 89 kb)

Additional file 3: Comparison of chemosensory proteins identified in our study with previous work. (XLSX 24 kb)

Additional file 4 **Table S3.** Primers used in the qPCR analysis. (XLSX 15 kb)

## Abbreviations

CO_2_: Carbon dioxide
CSP: Chemosensory protein
FPKM: Fragments per kb per million fragments
GO: Gene ontology
GOBP: General odorant binding protein
GR: Glutamate receptor
iGluR: Ionotropic glutamate receptor
IR: Ionotropic receptor
JTT: Jones-Taylor-Thornton amino acid substitution model
OBP: Odorant binding protein
OR: Odorant receptor
ORF: Open reading frame
PBP: Pheromone binding protein
PR: Pheromone receptor
SNMP: Sensory neuron membrane protein
TMD: Transmembrane domain
