# Supplementary material for: Identification of candidate chemosensory genes in Mythimna separata by transcriptomic analysis
Source: BMC Genomics. 2018 Jul 4;19:518. doi: 10.1186/s12864-018-4898-0 (PMC6030794; doi:10.1186/s12864-018-4898-0)
Supplement: Supplementary file 1 — Table S1. Assembly summary of M. separata antennal transcriptome. (DOCX 18 kb) [file 12864_2018_4898_MOESM1_ESM.docx]

Table S1. Assembly summary of *M. separata* transcriptome

|  | Sample | Total Number | Total  Length (nt) | Mean  Length (nt) | N50 | Consensus Sequences | | Distinct Clusters | Distinct Singletons |
| --- | --- | --- | --- | --- | --- | --- | --- | --- | --- |
| Contig | FA | 145,471 | 46,156,971 | 317 | 599 | - | - | | - |
|  | MA | 141,267 | 44,916,279 | 318 | 600 | - | - | | - |
|  | LP | 116,454 | 37,087,331 | 318 | 592 | - | - | | - |
|  | P | 128,834 | 39,486,244 | 306 | 544 | - | - | | - |
| Unigene | FA | 73,342 | 50,134,565 | 684 | 1,425 | 73,342 | 23,476 | | 49,866 |
|  | MA | 71,522 | 49,296,796 | 689 | 1,433 | 71,522 | 22,807 | | 48,715 |
|  | LP | 56,263 | 35,617,388 | 633 | 1,239 | 56,263 | 14,722 | | 41,541 |
|  | P | 64,136 | 40,833,173 | 637 | 1,245 | 64,136 | 18,456 | | 45,680 |
| Merge |  | 71,008 | 65,592,380 | 924 | 1,748 | 71,008 | 29,388 | | 41,620 |

Note: FA: Female antennae; MA: Male antennae; LP: Labial palp; P: Proboscis.
