# Supplementary material for: Identification of candidate chemosensory genes in Mythimna separata by transcriptomic analysis
Source: BMC Genomics. 2018 Jul 4;19:518. doi: 10.1186/s12864-018-4898-0 (PMC6030794; doi:10.1186/s12864-018-4898-0)
Supplement: Supplementary file 2 — Table S2. Candidate chemosensory genes of M. separata antennal transcriptome. Table S2–1: Unigenes of candidate odorant receptors with gene name, length, ORF, best blastx hit and identity, etc. Table S2–2: Unigenes of candidate ionotropic receptors. Table S2–3: Unigenes of candidate gustatory receptors. Table S2–4: Unigenes of candidate odorant binding proteins. Table S2–5: Unigenes of candidate chemosensory proteins. Table S2–6: Unigenes of candidate sensory neuron membrane proteins. (DOCX 89 kb) [file 12864_2018_4898_MOESM2_ESM.docx]

Table S2-1. Unigenes of candidate olfactory receptors in *M. separate*

| Gene name | Unigene reference | Length (nt) | ORF (aa) | Status | TMD (No.) | Evalue | BLASTx best hit | FA  FPKM | MA FPKM | P  FPKM | LP FPKM |
| --- | --- | --- | --- | --- | --- | --- | --- | --- | --- | --- | --- |
| MsepOrco | CL9933.Contig3 | 3036 | 473 | Complete ORF | 7 | 0 | dbj\|BAG71415.1\| olfactory receptor-2 [Mythimna separata] | 26.10 | 28.87 | 0.36 | 0.12 |
| MsepPR1 | CL2288.Contig2 | 1587 | 432 | Complete ORF | 3 | 0 | gb\|AGY14579.2\| putative odorant receptor [Sesamia inferens] | 0.42 | 8.96 | 0.12 | 0.02 |
| MsepPR2 | Unigene15043 | 1837 | 435 | Complete ORF | 6 | 0 | gb\|AGS41442.1\| olfactory receptor 3 [Agrotis segetum] | 50.93 | 56.32 | 0.43 | 1.16 |
| MsepPR3 | Unigene3409 | 1623 | 424 | Complete ORF | 6 | 0 | dbj\|BAG71423.2\| olfactory receptor [Mythimna separata] | 7.48 | 260.07 | 1.71 | 1.13 |
| MsepPR4 | Unigene33066 | 1463 | 445 | Complete ORF | 4 | 0 | dbj\|BAG71414.1\| olfactory receptor-1 [Mythimna separata] | 0.42 | 18.56 | 0.16 | 0.02 |
| MsepPR5 | Unigene29562 | 1441 | 431 | Complete ORF | 6 | 1e-166 | gb\|ACL81182.1\| putative olfactory receptor 16 [Spodoptera littoralis] | 7.52 | 0.20 | 0.06 | 0.02 |
| MsepPR6 | Unigene33223 | 1620 | 434 | Complete ORF | 8 | 3e-74 | gb\|AGI96751.1\| olfactory receptor 16 [Spodoptera litura] | 0.42 | 6.29 | 0.00 | 0.07 |
| MsepOR1 | CL11184.Contig1 | 1499 | 452 | Complete ORF | 4 | 0 | gb\|AGG08878.1\| putative olfactory receptor 12 [Spodoptera litura] | 3.55 | 2.60 | 0.16 | 0.39 |
| MsepOR2 | CL1213.Contig4 | 1810 | 440 | Complete ORF | 6 | 0 | gb\|AIG51888.1\| odorant receptor [Helicoverpa armigera] | 13.67 | 9.68 | 0.02 | 0.04 |
| MsepOR3 | Unigene18337 | 1460 | 429 | Complete ORF | 6 | 0 | gb\|AIG51891.1\| odorant receptor, partial [Helicoverpa armigera] | 14.18 | 6.16 | 0.23 | 0.15 |
| MsepOR4 | Unigene28126 | 1446 | 429 | Complete ORF | 6 | 0 | gb\|AGG08877.1\| putative olfactory receptor 44 [Spodoptera litura] | 7.81 | 6.53 | 0.18 | 0.30 |
| MsepOR5 | Unigene4204 | 1621 | 424 | Complete ORF | 6 | 1e-143 | dbj\|BAR43471.1\| putative olfactory receptor 29 [Ostrinia furnacalis] | 5.98 | 3.03 | 0.02 | 0.13 |
| MsepOR6 | CL604.Contig2 | 1629 | 422 | Complete ORF | 5 | 1e-147 | dbj\|BAR43488.1\| putative olfactory receptor 46 [Ostrinia furnacalis] | 1.18 | 0.60 | 0.03 | 0.02 |
| MsepOR7 | CL11003.Contig1 | 1417 | 420 | Complete ORF | 7 | 0 | gb\|AIG51890.1\| odorant receptor [Helicoverpa armigera] | 11.68 | 9.28 | 0.00 | 0.03 |
| MsepOR8 | Unigene23410 | 1553 | 419 | Complete ORF | 6 | 0 | gb\|AIG51898.1\| odorant receptor [Helicoverpa armigera] | 39.53 | 30.50 | 0.06 | 0.06 |
| MsepOR9 | Unigene1824 | 1516 | 414 | Complete ORF | 6 | 1e-136 | gb\|AFL70813.1\| odorant receptor 50, partial [Manduca sexta] | 5.55 | 4.43 | 1.13 | 0.63 |
| MsepOR10 | Unigene19092 | 1546 | 412 | Complete ORF | 6 | 1e-165 | dbj\|BAR43458.1\| putative olfactory receptor 16 [Ostrinia furnacalis] | 6.46 | 5.33 | 0.04 | 0.02 |
| MsepOR11 | Unigene24866 | 1310 | 412 | Complete ORF | 7 | 0 | gb\|AEF32141.1\| odorant receptor [Spodoptera exigua] | 7.87 | 4.67 | 0.34 | 0.16 |
| MsepOR12 | CL9055.Contig2 | 1309 | 412 | Complete ORF | 4 | 0 | gb\|AIG51871.1\| odorant receptor [Helicoverpa armigera] | 4.37 | 2.69 | 0.16 | 0.02 |
| MsepOR13 | CL4724.Contig1 | 1906 | 408 | Complete ORF | 4 | 0 | gb\|AIG51875.1\| odorant receptor [Helicoverpa armigera] | 5.23 | 4.79 | 0.02 | 0.02 |
| MsepOR14 | CL533.Contig1 | 1282 | 408 | 5' lost | 6 | 0 | gb\|AIG51860.1\| odorant receptor [Helicoverpa armigera] | 7.11 | 4.94 | 0.07 | 0.03 |
| MsepOR15 | Unigene2916 | 1675 | 407 | Complete ORF | 5 | 0 | gb\|AGK90001.1\| olfactory receptor 7 [Helicoverpa armigera] | 8.56 | 5.92 | 0.04 | 0.06 |
| MsepOR16 | CL2932.Contig1 | 1375 | 407 | Complete ORF | 6 | 1e-135 | gb\|AIG51882.1\| odorant receptor, partial [Helicoverpa armigera] | 4.10 | 3.39 | 0.22 | 0.20 |
| MsepOR17 | Unigene13411 | 1276 | 407 | Complete ORF | 6 | 1e-130 | ref\|XP_012545317.1\| PREDICTED: odorant receptor 4-like [Bombyx mori] | 6.94 | 5.91 | 0.17 | 0.38 |
| MsepOR18 | Unigene13562 | 1365 | 406 | Complete ORF | 7 | 0 | gb\|AIG51896.1\| odorant receptor, partial [Helicoverpa armigera] | 8.02 | 8.54 | 0.00 | 0.00 |
| MsepOR19 | Unigene16291 | 1569 | 404 | Complete ORF | 5 | 0 | gb\|AII01090.1\| odorant receptor [Dendrolimus kikuchii] | 6.46 | 2.79 | 0.00 | 0.06 |
| MsepOR20 | CL5088.Contig1 | 1572 | 402 | Complete ORF | 6 | 0 | gb\|AIZ00994.1\| putative olfactory receptor 9 [Helicoverpa armigera] | 2.79 | 2.36 | 0.00 | 0.09 |
| MsepOR21 | Unigene10595 | 1449 | 402 | Complete ORF | 5 | 0 | gb\|AJD81557.1\| olfactory receptor 21, partial [Helicoverpa assulta] | 17.50 | 12.42 | 3.61 | 0.02 |
| MsepOR22 | Unigene16505 | 1423 | 402 | Complete ORF | 6 | 1e-180 | gb\|AGY14593.1\| putative odorant receptor [Sesamia inferens] | 13.53 | 0.53 | 0.11 | 0.11 |
| MsepOR23 | Unigene11935 | 2079 | 401 | Complete ORF | 6 | 1e-179 | ref\|NP_001166613.1\| olfactory receptor 22 [Bombyx mori] | 3.14 | 2.06 | 0.02 | 0.02 |
| MsepOR24 | Unigene13321 | 1530 | 401 | Complete ORF | 5 | 0 | gb\|AIG51886.1\| odorant receptor [Helicoverpa armigera] | 4.23 | 3.33 | 0.00 | 0.05 |
| MsepOR25 | CL8257.Contig1 | 1845 | 398 | Complete ORF | 5 | 0 | gb\|ACL81188.1\| putative olfactory receptor 18 [Mamestra brassicae] | 12.39 | 5.05 | 0.05 | 0.00 |
| MsepOR26 | Unigene16237 | 1843 | 397 | Complete ORF | 7 | 3e -78 | dbj\|BAR43481.1\| putative olfactory receptor 39 [Ostrinia furnacalis] | 1.58 | 1.43 | 0.09 | 0.09 |
| MsepOR27 | Unigene24998 | 1389 | 397 | Complete ORF | 6 | 0 | gb\|AIG51879.1\| odorant receptor [Helicoverpa armigera] | 18.77 | 15.01 | 8.27 | 3.04 |
| MsepOR28 | CL916.Contig1 | 4450 | 397 | Complete ORF | 6 | 0 | dbj\|BAR43469.1\| putative olfactory receptor 27 [Ostrinia furnacalis] | 2.34 | 1.99 | 0.29 | 0.28 |
| MsepOR29 | Unigene10728 | 1226 | 396 | 3'lost | 6 | 0 | gb\|AII01083.1\| odorant receptor [Dendrolimus kikuchii] | 16.06 | 10.98 | 0.05 | 0.03 |
| MsepOR30 | Unigene22101 | 1506 | 395 | Complete ORF | 6 | 0 | gb\|AIZ00995.1\| putative olfactory receptor 29 [Helicoverpa armigera] | 9.47 | 7.87 | 0.11 | 0.04 |
| MsepOR31 | Unigene4980 | 1349 | 395 | Complete ORF | 6 | 1e-156 | tpg\|DAA05986.1\| TPA_exp: odorant receptor 30 [Bombyx mori] | 5.96 | 5.03 | 0.02 | 0.05 |
| MsepOR32 | CL5979.Contig1 | 1386 | 393 | Complete ORF | 7 | 0 | gb\|AIG51868.1\| odorant receptor [Helicoverpa armigera] | 30.21 | 14.31 | 0.02 | 0.16 |
| MsepOR33 | CL9419.Contig2 | 1301 | 393 | 5' lost | 6 | 0 | dbj\|BAR43462.1\| putative olfactory receptor 20 [Ostrinia furnacalis] | 1.47 | 1.56 | 0.21 | 0.16 |
| MsepOR34 | CL4039.Contig2 | 2010 | 392 | Complete ORF | 7 | 1e-115 | tpg\|DAA05974.1\| TPA_exp: odorant receptor 15 [Bombyx mori] | 0.66 | 0.42 | 0.06 | 0.02 |
| MsepOR35 | CL3525.Contig2 | 1651 | 391 | Complete ORF | 7 | 0 | gb\|AIG51856.1\| odorant receptor [Helicoverpa armigera] | 2.13 | 1.86 | 0.01 | 0.04 |
| MsepOR36 | CL3525.Contig5 | 1657 | 390 | Complete ORF | 7 | 0 | gb\|AIG51856.1\| odorant receptor [Helicoverpa armigera] | 0.74 | 0.61 | 0.00 | 0.02 |
| MsepOR37 | CL3525.Contig1 | 1648 | 390 | Complete ORF | 7 | 0 | gb\|AIG51856.1\| odorant receptor [Helicoverpa armigera] | 2.82 | 3.04 | 0.00 | 0.00 |
| MsepOR38 | CL2497.Contig1 | 1568 | 391 | Complete ORF | 4 | 0 | gb\|AIG51873.1\| odorant receptor [Helicoverpa armigera] | 2.26 | 1.76 | 0.00 | 0.10 |
| MsepOR39 | CL3525.Contig3 | 1425 | 391 | Complete ORF | 7 | 0 | gb\|AIG51856.1\| odorant receptor [Helicoverpa armigera] | 0.88 | 0.67 | 0.10 | 0.14 |
| MsepOR40 | Unigene13634 | 1468 | 390 | Complete ORF | 5 | 0 | gb\|AGK90020.1\| olfactory receptor 17 [Helicoverpa assulta] | 3.62 | 4.88 | 0.00 | 0.00 |
| MsepOR41 | Unigene21956 | 1457 | 390 | Complete ORF | 6 | 1e-167 | gb\|AIG51885.1\| odorant receptor [Helicoverpa armigera] | 6.06 | 5.07 | 0.13 | 0.21 |
| MsepOR42 | Unigene19260 | 1523 | 388 | Complete ORF | 5 | 0 | gb\|AGK90017.1\| olfactory receptor 10 [Helicoverpa assulta] | 4.10 | 3.72 | 0.16 | 0.02 |
| MsepOR43 | Unigene7276 | 1280 | 387 | Complete ORF | 6 | 1e-163 | gb\|AII01102.1\| odorant receptor [Dendrolimus kikuchii] | 6.90 | 4.31 | 0.02 | 0.05 |
| MsepOR44 | Unigene1808 | 1272 | 385 | Complete ORF | 6 | 0 | ref\|NP_001104832.2\| olfactory receptor 16 [Bombyx mori] | 5.32 | 4.28 | 0.06 | 0.06 |
| MsepOR45 | Unigene7953 | 1241 | 385 | 5' lost | 5 | 0 | gb\|AIG51892.1\| odorant receptor [Helicoverpa armigera] | 8.82 | 8.07 | 0.04 | 0.17 |
| MsepOR46 | CL10654.Contig1 | 1246 | 373 | 5' lost | 6 | 0 | gb\|AIG51887.1\| odorant receptor [Helicoverpa armigera] | 3.64 | 2.26 | 0.00 | 0.04 |
| MsepOR47 | CL4039.Contig1 | 1969 | 372 | Complete ORF | 6 | 2e-96 | ref\|NP_001091789.1\| olfactory receptor 15 [Bombyx mori] | 0.94 | 0.68 | 0.02 | 0.04 |
| MsepOR48 | Unigene1417 | 1189 | 349 | 5' lost | 6 | 1e -114 | gb\|AJF23822.1\| olfactory receptor OR67, partial [Planotortrix octo] | 2.71 | 2.61 | 0.01 | 0.00 |
| MsepOR49 | Unigene16191 | 1272 | 337 | 3' lost | 5 | 1e-131 | dbj\|BAR43488.1\| putative olfactory receptor 46 [Ostrinia furnacalis] | 5.52 | 3.14 | 0.02 | 0.00 |
| MsepOR50 | Unigene25861 | 1220 | 336 | 5' lost | 5 | 1e-138 | gb\|AII01092.1\| odorant receptor [Dendrolimus kikuchii] | 2.52 | 2.00 | 0.02 | 0.02 |
| MsepOR51 | Unigene25078 | 1069 | 322 | 5' lost | 4 | 1e-180 | gb\|AIG51906.1\| odorant receptor [Helicoverpa armigera] | 4.70 | 4.27 | 0.17 | 0.04 |
| MsepOR52 | Unigene4931 | 782 | 201 | 5' lost | 2 | 1e-121 | gb\|AGK89999.1\| olfactory receptor 3 [Helicoverpa armigera] | 3.56 | 2.07 | 0.02 | 0.00 |
| MsepOR53 | Unigene29326 | 614 | 187 | 5' lost | 2 | 3e-69 | dbj\|BAR43494.1\| putative olfactory receptor 52 [Ostrinia furnacalis] | 2.88 | 0.00 | 0.03 | 0.00 |
| MsepOR54 | Unigene39385 | 767 | 177 | 5' lost | 2 | 1e -75 | gb\|ACC63237.1\| olfactory receptor 9 [Helicoverpa armigera] | 0.19 | 0.17 | 0.00 | 0.00 |
| MsepOR55 | Unigene29331 | 608 | 174 | 5' lost | 0 | 4e-75 | gb\|AIG51902.1\| odorant receptor, partial [Helicoverpa armigera] | 3.15 | 0.05 | 2.52 | 0.07 |

Note: FA: Female antennae; MA: Male antennae; P: Proboscis; LP: Labial palp.

Table S2-2. Unigenes of candidate ionotropic receptors in *M. separate*

| Gene name | Unigene reference | Length (nt) | ORF (aa) | Status | TMD (No.) | Evalue | BLASTx best hit | FA  FPKM | MA FPKM | P  FPKM | LP FPKM |
| --- | --- | --- | --- | --- | --- | --- | --- | --- | --- | --- | --- |
| MsepIR1 | Unigene22320 | 2136 | 658 | Complete ORF | 3 | 0 | gb\|ADR64688.1\| putative chemosensory ionotropic receptor IR1 [Spodoptera littoralis] | 6.87 | 6.14 | 0.05 | 0.08 |
| MsepIR1.1 | CL5211.Contig1 | 2212 | 651 | Complete ORF | 3 | 1e-126 | dbj\|BAR64812.1\| ionotropic receptor, partial [Ostrinia furnacalis] | 26.27 | 27.98 | 0.23 | 0.17 |
| MsepIR4 | CL4582.Contig3 | 2239 | 608 | Complete ORF | 1 | 1e-132 | dbj\|BAR64813.1\| ionotropic receptor, partial [Ostrinia furnacalis] | 8.88 | 6.30 | 0.11 | 0.01 |
| MsepIR7d.1 | Unigene9040 | 2739 | 595 | Complete ORF | 4 | 1e-141 | gb\|AIG51916.1\| ionotropic receptor, partial [Helicoverpa armigera] | 3.65 | 3.16 | 1.17 | 0.07 |
| MsepIR7d.2 | Unigene18157 | 730 | 243 | 5', 3' lost | 2 | 3e-91 | gb\|AIG51917.1\| ionotropic receptor, partial [Helicoverpa armigera] | 3.14 | 1.48 | 0.22 | 0.00 |
| MsepIR8a | CL3846.Contig1 | 3327 | 899 | Complete ORF | 4 | 0 | dbj\|BAR64796.1\| ionotropic receptor [Ostrinia furnacalis] | 114.31 | 73.11 | 0.65 | 0.78 |
| MsepIR21a | CL4768.Contig2 | 8624 | 854 | Complete ORF | 3 | 0 | gb\|ADR64678.1\| putative chemosensory ionotropic receptor IR21a [Spodoptera littoralis] | 6.06 | 4.74 | 0.69 | 0.42 |
| MsepIR25a | Unigene6676 | 3101 | 925 | Complete ORF | 3 | 0 | gb\|AJD81628.1\| ionotropic receptor 25a, partial [Helicoverpa assulta] | 141.59 | 135.55 | 7.74 | 11.58 |
| MsepIR41a | Unigene23832 | 1950 | 614 | Complete ORF | 3 | 0 | gb\|ADR64681.1\| putative chemosensory ionotropic receptor IR41a [Spodoptera littoralis] | 21.55 | 17.63 | 0.45 | 0.69 |
| MsepIR64a | CL507.Contig1 | 2034 | 475 | 5' lost | 2 | 1e-173 | dbj\|BAR64801.1\| ionotropic receptor [Ostrinia furnacalis] | 5.72 | 4.33 | 0.29 | 0.19 |
| MsepIR68a | Unigene15362 | 917 | 305 | 5', 3' lost | 3 | 0 | gb\|ADR64682.1\| putative chemosensory ionotropic receptor IR68a [Spodoptera littoralis] | 3.55 | 1.73 | 0.00 | 0.00 |
| MsepIR75d | Unigene20198 | 963 | 189 | 5' lost | 0 | 1e-75 | dbj\|BAR64804.1\| ionotropic receptor, partial [Ostrinia furnacalis] | 2.05 | 2.57 | 0.83 | 1.11 |
| MsepIR75p | CL3280.Contig2 | 1890 | 619 | 5' lost | 3 | 0 | gb\|ADR64684.1\| putative chemosensory ionotropic receptor IR75p [Spodoptera littoralis] | 3.76 | 3.20 | 0.03 | 0.01 |
| MsepIR75p.1 | CL3280.Contig1 | 2279 | 617 | Complete ORF | 3 | 0 | gb\|ADR64684.1\| putative chemosensory ionotropic receptor IR75p [Spodoptera littoralis] | 5.16 | 4.76 | 0.06 | 0.02 |
| MsepIR75p.2 | CL3280.Contig4 | 740 | 235 | 5' lost | 2 | 1e-106 | dbj\|BAR64805.1\| ionotropic receptor [Ostrinia furnacalis] | 9.50 | 8.18 | 0.25 | 0.07 |
| MsepIR75q.1 | CL7702.Contig1 | 2199 | 628 | Complete ORF | 3 | 0 | gb\|ADR64685.1\| putative chemosensory ionotropic receptor IR75q.2 [Spodoptera littoralis] | 24.59 | 23.24 | 0.37 | 0.29 |
| MsepIR75q.2 | Unigene27730 | 2291 | 368 | 5' lost | 4 | 0 | gb\|ADR64685.1\| putative chemosensory ionotropic receptor IR75q.2 [Spodoptera littoralis] | 20.31 | 19.25 | 0.06 | 0.20 |
| MsepIR76b | CL9451.Contig1 | 2034 | 544 | Complete ORF | 3 | 0 | gb\|AGY49253.1\| putative ionotropic receptor [Sesamia inferens] | 97.85 | 80.20 | 10.37 | 1.76 |
| MsepIR87a | Unigene11067 | 2187 | 651 | Complete ORF | 3 | 0 | dbj\|BAR64810.1\| ionotropic receptor [Ostrinia furnacalis] | 8.26 | 8.33 | 0.06 | 0.09 |
| MsepIR93a | Unigene9362 | 2864 | 872 | Complete ORF | 3 | 0 | dbj\|BAR64811.1\| ionotropic receptor [Ostrinia furnacalis] | 7.17 | 5.38 | 0.05 | 0.15 |

Note: FA: Female antennae; MA: Male antennae; P: Proboscis; LP: Labial palp.

Table S2-3. Unigenes of candidate gustatory receptors in *M. separate*

| Gene name | Unigene reference | Length (nt) | ORF (aa) | Status | TMD (No.) | Evalue | BLASTx best hit | FA  FPKM | MA  FPKM | P  FPKM | LP FPKM |
| --- | --- | --- | --- | --- | --- | --- | --- | --- | --- | --- | --- |
| MsepGR1 | Unigene28441 | 1807 | 501 | Complete ORF | 7 | 0 | gb\|AIG51907.1\| gustatory receptor [Helicoverpa armigera] | 0.37 | 0.26 | 0.09 | 52.34 |
| MsepGR2 | Unigene20603 | 1548 | 434 | Complete ORF | 7 | 0 | gb\|AIG51908.1\| gustatory receptor [Helicoverpa armigera] | 0.11 | 0.23 | 0.72 | 51.30 |
| MsepGR3 | CL939.Contig1 | 2101 | 475 | Complete ORF | 8 | 0 | gb\|AIG51909.1\| gustatory receptor [Helicoverpa armigera] | 1.04 | 0.87 | 0.33 | 7.28 |
| MsepGR4 | Unigene20551 | 1658 | 476 | Complete ORF | 6 | 0 | gb\|AGA04648.1\| gustatory receptor [Helicoverpa armigera] | 3.15 | 3.01 | 0.73 | 0.16 |
| MsepGR5 | Unigene10656 | 868 | 289 | 5', 3' lost | 5 | 1e-119 | gb\|AIG51912.1\| gustatory receptor, partial [Helicoverpa armigera] | 1.39 | 1.47 | 5.00 | 0.14 |
| MsepGR6 | Unigene33938 | 644 | 214 | 5', 3' lost | 4 | 0 | ref\|XP_012552784.1\| PREDICTED: gustatory receptor for sugar taste 64f-like [Bombyx mori] | 1.05 | 2.00 | 0.24 | 0.00 |
| MsepGR7 | Unigene18757 | 383 | 127 | 5', 3' lost | 0 | 3e-26 | tpg\|DAA06395.1\| TPA_inf: gustatory receptor 63 [Bombyx mori] | 2.12 | 1.90 | 0.76 | 0.14 |
| MsepGR8 | Unigene38709 | 407 | 122 | 5' lost | 2 | 2e-58 | gb\|AIG51911.1\| gustatory receptor [Helicoverpa armigera] | 0.29 | 0.23 | 3.26 | 0.07 |
| MsepGR9 | Unigene40038 | 592 | 121 | 3' lost | 2 | 3e-02 | ref\|XP_008551737.1\| PREDICTED: putative gustatory receptor 28b [Microplitis demolitor] | 0.05 | 0.00 | 1.57 | 0.00 |
| MsepGR10 | Unigene40834 | 341 | 112 | 5', 3' lost | 3 | 2e-61 | gb\|AGK90023.1\| gustatory receptor 1 [Helicoverpa assulta] | 0.60 | 0.65 | 0.86 | 0.08 |
| MsepGR11 | Unigene40316 | 316 | 102 | 3' lost | 1 | 7e-41 | gb\|AIG51911.1\| gustatory receptor [Helicoverpa armigera] | 0.09 | 0.00 | 1.59 | 0.00 |
| MsepGR12 | Unigene40362 | 273 | 90 | 5', 3' lost | 2 | 3e-39 | gb\|AJD81598.1\| gustatory receptor 5, partial [Helicoverpa assulta] | 0.00 | 0.00 | 1.84 | 0.00 |
| MsepGR13 | Unigene29970 | 274 | 82 | 5', 3' lost | 1 | 2e-08 | gb\|AGK90012.1\| gustatory receptor 5 [Helicoverpa armigera] | 2.23 | 0.46 | 0.29 | 0.20 |
| MsepGR14 | Unigene31341 | 229 | 75 | 5', 3' lost | 1 | 2e -26 | gb\|AJD81597.1\| gustatory receptor 4, partial [Helicoverpa assulta] | 0.63 | 0.42 | 0.12 | 0.00 |
| MsepGR15 | Unigene40624 | 226 | 74 | 5', 3' lost | 1 | 1e-33 | gb\|AJD81600.1\| gustatory receptor 7, partial [Helicoverpa assulta] | 0.77 | 0.70 | 1.52 | 0.00 |
| MsepGR16 | CL1521.Contig1 | 218 | 72 | 5', 3' lost | 2 | 1e-02 | emb\|CAL23152.1\| gustatory receptor candidate 19 [Tribolium castaneum] | 3.51 | 2.66 | 0.20 | 0.54 |

Note: FA: Female antennae; MA: Male antennae; P: Proboscis; LP: Labial palp.

Table S2-4. Unigenes of candidate odorant binding proteins in *M. separate*

| Gene name | Unigene reference | Length (nt) | ORF (aa) | Status | Singal peptide | Evalue | BLASTx best hit | FA  FPKM | MA FPKM | P  FPKM | LP FPKM |
| --- | --- | --- | --- | --- | --- | --- | --- | --- | --- | --- | --- |
| MsepGOBP1 | CL8558.Contig4 | 1168 | 101 | 5' lost | N | 4e -52 | gb\|ABI24159.1\| general odorant binding protein 1, partial [Agrotis segetum] | 1151.23 | 666.17 | 3.81 | 10.62 |
| MsepGOBP2 | Unigene7217 | 781 | 162 | Complete ORF | Y | 2e-95 | gb\|AKI87961.1\| general odorant binding protein 2 [Spodoptera litura] | 49665.66 | 40811.71 | 184.57 | 301.50 |
| MsepPBP1 | CL5079.Contig2 | 1109 | 170 | Complete ORF | Y | 1e-105 | dbj\|BAG71416.1\| pheromone binding protein [Mythimna separata] | 3964.28 | 8106.27 | 35.97 | 39.27 |
| MsepPBP2 | CL2.Contig6 | 1399 | 165 | Complete ORF | Y | 2e-95 | gb\|AEB54583.1\| PBP2 [Helicoverpa armigera] | 2530.63 | 8426.37 | 89.53 | 28.27 |
| MsepPBP3 | Unigene9512 | 607 | 141 | 3' lost | Y | 7e-85 | gb\|AFM36758.1\| pheromone-binding protein 3 [Agrotis ipsilon] | 10528.23 | 3848.45 | 16.20 | 48.25 |
| MsepOBP1 | CL7152.Contig1 | 1130 | 334 | Complete ORF | Y | 7e-68 | ref\|XP_004927370.1\| PREDICTED: general odorant-binding protein 71 [Bombyx mori] | 23.28 | 18.70 | 125.66 | 46.25 |
| MsepOBP2 | Unigene14790 | 1083 | 247 | Complete ORF | Y | 1e-92 | gb\|AKI87962.1\| odorant binding protein 1 [Spodoptera litura] | 378.46 | 376.78 | 2.40 | 2.81 |
| MsepOBP3 | Unigene10043 | 782 | 197 | Complete ORF | Y | 3e-82 | gb\|AGC92793.1\| odorant-binding protein 19 [Helicoverpa assulta] | 40.29 | 102.07 | 26.27 | 116.74 |
| MsepOBP4 | CL7075.Contig2 | 642 | 183 | Complete ORF | Y | 4e-96 | gb\|AGR39564.1\| odorant binding protein 1, partial [Agrotis ipsilon] | 63.59 | 70.53 | 76.84 | 15.22 |
| MsepOBP5 | CL5174.Contig3 | 692 | 168 | Complete ORF | Y | 3e-73 | gb\|AKI87965.1\| odorant binding protein 4 [Spodoptera litura] | 8409.49 | 7232.22 | 190.53 | 74.96 |
| MsepOBP6 | CL5042.Contig1 | 1163 | 165 | Complete ORF | Y | 4e-10 | gb\|AII01009.1\| odorant binding protein [Dendrolimus kikuchii] | 895.14 | 946.56 | 1282.32 | 47.45 |
| MsepOBP7 | CL6893.Contig2 | 628 | 153 | Complete ORF | Y | 2e-12 | gb\|AFI57167.1\| odorant-binding protein 18 [Helicoverpa armigera] | 14.24 | 15.99 | 174.11 | 4.01 |
| MsepOBP8 | CL8135.Contig1 | 619 | 149 | Complete ORF | Y | 2e-59 | gb\|AEB54581.1\| OBP5 [Helicoverpa armigera] | 0.84 | 0.31 | 32.80 | 0.53 |
| MsepOBP9 | CL11163.Contig1 | 596 | 149 | Complete ORF | Y | 7e-59 | gb\|AEB54581.1\| OBP5 [Helicoverpa armigera] | 614.23 | 474.87 | 365.85 | 151.52 |
| MsepOBP10 | Unigene24324 | 670 | 148 | Complete ORF | Y | 3e-65 | gb\|AEB54591.1\| OBP7 [Helicoverpa armigera] | 7100.50 | 6725.50 | 1746.18 | 1242.58 |
| MsepOBP11 | Unigene38891 | 573 | 148 | Complete ORF | Y | 3e-51 | gb\|AEB54581.1\| OBP5 [Helicoverpa armigera] | 0.35 | 0.17 | 4.21 | 0.10 |
| MsepOBP12 | Unigene10077 | 653 | 147 | Complete ORF | Y | 2e-58 | gb\|AFM77984.1\| oderant binding protein 6 [Spodoptera exigua] | 12.05 | 10.53 | 199.80 | 29.01 |
| MsepOBP13 | Unigene38448 | 524 | 147 | Complete ORF | Y | 3e-50 | gb\|AFI57167.1\| odorant-binding protein 18 [Helicoverpa armigera] | 0.28 | 0.24 | 3.34 | 0.00 |
| MsepOBP14 | CL10075.Contig1 | 830 | 146 | Complete ORF | Y | 3e-74 | gb\|AFG72998.1\| odorant-binding protein 1 [Cnaphalocrocis medinalis] | 943.77 | 810.71 | 4.95 | 8.01 |
| MsepOBP15 | CL11163.Contig2 | 584 | 146 | Complete ORF | Y | 4e-83 | gb\|AAL66739.1\|AF461143_1 pheromone binding protein 4 [Mamestra brassicae] | 1338.24 | 1492.84 | 1015.26 | 1676.85 |
| MsepOBP16 | Unigene5414 | 544 | 146 | Complete ORF | Y | 4e-83 | gb\|AGR39569.1\| odorant binding protein 6, partial [Agrotis ipsilon] | 0.27 | 0.06 | 1.12 | 2.92 |
| MsepOBP17 | Unigene27792 | 548 | 146 | Complete ORF | Y | 8e-25 | gb\|AAL66739.1\|AF461143_1 pheromone binding protein 4 [Mamestra brassicae] | 6.15 | 19.08 | 3.63 | 0.35 |
| MsepOBP18 | Unigene25044 | 805 | 145 | Complete ORF | Y | 2e-23 | gb\|AGS36753.1\| OBP13 [Sesamia inferens] | 0.18 | 0.47 | 91.97 | 0.24 |
| MsepOBP19 | Unigene27116 | 499 | 145 | Complete ORF | Y | 5e-83 | gb\|ADY17886.1\| odorant binding protein [Spodoptera exigua] | 10.01 | 9.59 | 1.91 | 2.25 |
| MsepOBP20 | CL7207.Contig1 | 1046 | 143 | Complete ORF | Y | 6e-43 | gb\|AGC92789.1\| odorant-binding protein 9 [Helicoverpa assulta] | 2204.45 | 1850.62 | 334.63 | 18.79 |
| MsepOBP21 | Unigene7260 | 576 | 143 | 5' lost | Y | 6e-91 | gb\|AGH70104.1\| odorant binding protein 8 [Spodoptera exigua] | 24840.54 | 8491.00 | 108.47 | 154.88 |
| MsepOBP22 | CL1928.Contig2 | 3166 | 142 | Complete ORF | Y | 5e-76 | gb\|AGS36747.1\| OBP5, partial [Sesamia inferens] | 92.73 | 149.86 | 1.94 | 2.04 |
| MsepOBP23 | Unigene38901 | 460 | 141 | 3' lost | Y | 1e-28 | gb\|AGR39565.1\| odorant binding protein 2 [Agrotis ipsilon] | 0.38 | 0.14 | 7.49 | 0.36 |
| MsepOBP24 | CL4633.Contig2 | 698 | 141 | Complete ORF | Y | 5e-26 | gb\|AGP03459.1\| SexiOBP13 [Spodoptera exigua] | 0.04 | 0.00 | 7.82 | 0.78 |
| MsepOBP25 | CL4633.Contig1 | 676 | 139 | Complete ORF | Y | 4e-26 | gb\|AGP03459.1\| SexiOBP13 [Spodoptera exigua] | 0.04 | 0.05 | 19.25 | 1.70 |
| MsepOBP26 | Unigene1386 | 561 | 139 | Complete ORF | Y | 6e-86 | gb\|AEB54589.1\| OBP8 [Helicoverpa armigera] | 2286.55 | 562.05 | 2.32 | 9.66 |
| MsepOBP27 | CL5040.Contig3 | 856 | 138 | Complete ORF | Y | 2e-59 | gb\|AEX07280.1\| odorant-binding protein [Helicoverpa armigera] | 174.60 | 243.01 | 11.52 | 136.29 |
| MsepOBP28 | CL966.Contig5 | 2514 | 137 | Complete ORF | Y | 1e-49 | gb\|AKI87964.1\| odorant binding protein 3 [Spodoptera litura] | 1530.54 | 5400.68 | 46.84 | 141.53 |
| MsepOBP29 | Unigene15224 | 825 | 133 | Complete ORF | Y | 1e-79 | gb\|AGH70105.1\| odorant binding protein 9 [Spodoptera exigua] | 706.77 | 718.15 | 27.15 | 49.04 |
| MsepOBP30 | Unigene10689 | 567 | 132 | Complete ORF | N | 1e-73 | gb\|AGS36748.1\| OBP6, partial [Sesamia inferens] | 3.53 | 4.25 | 1.17 | 0.24 |
| MsepOBP31 | Unigene996 | 444 | 130 | 5' lost | N | 3e-78 | gb\|AGP03460.1\| SexiOBP14 [Spodoptera exigua] | 1.90 | 1.36 | 0.54 | 0.86 |
| MsepOBP32 | Unigene9977 | 567 | 126 | 5' lost | N | 6e-06 | dbj\|BAI22689.1\| odorant binding protein [Bombyx mori] | 19.97 | 24.59 | 61.34 | 0.29 |
| MsepOBP33 | Unigene11478 | 386 | 74 | 5' lost | N | 7.e-30 | gb\|AGP03455.1\| SexiOBP9 [Spodoptera exigua] | 1.05 | 1.97 | 0.41 | 0.64 |

Note: FA: Female antennae; MA: Male antennae; P: Proboscis; LP: Labial palp.

Table S2-5. Unigenes of candidate chemosensory proteins in *M. separate*

| Gene name | Unigene reference | Length (nt) | ORF (aa) | Status | Singal peptide | Evalue | BLASTx best hit | FA  FPKM | MA FPKM | P  FPKM | LP FPKM |
| --- | --- | --- | --- | --- | --- | --- | --- | --- | --- | --- | --- |
| MsepCSP1 | CL10931.Contig1 | 2332 | 297 | Complete ORF | Y | 1e-141 | gb\|AIW65104.1\| chemosensory protein [Helicoverpa armigera] | 10.17 | 11.75 | 6.51 | 27.89 |
| MsepCSP2 | Unigene4126 | 707 | 156 | Complete ORF | Y | 9e-51 | gb\|AGR39578.1\| chemosensory protein 8 [Agrotis ipsilon] | 136.98 | 312.34 | 241.94 | 1329.44 |
| MsepCSP3 | CL2860.Contig1 | 1777 | 149 | Complete ORF | Y | 1e-77 | gb\|AGY49270.1\| putative chemosensory protein [Sesamia inferens] | 1209.73 | 1012.50 | 6.86 | 11.18 |
| MsepCSP4 | CL5692.Contig3 | 855 | 129 | Complete ORF | N | 7e-49 | gb\|AEX07267.1\| CSP6 [Helicoverpa armigera] | 105.69 | 150.54 | 18.69 | 46.26 |
| MsepCSP5 | Unigene18615 | 782 | 128 | Complete ORF | Y | 6e-61 | gb\|AAF71289.1\|AF255918_1 chemosensory protein [Mamestra brassicae] | 8141.57 | 7387.13 | 30035.48 | 3410.56 |
| MsepCSP6 | CL1786.Contig1 | 723 | 128 | Complete ORF | Y | 4e-69 | gb\|AAF71290.2\|AF255919_1 chemosensory protein [Mamestra brassicae] | 185.72 | 154.53 | 1758.67 | 1185.30 |
| MsepCSP7 | CL1786.Contig3 | 534 | 128 | Complete ORF | Y | 5e-68 | gb\|AGY49255.1\| putative chemosensory protein [Sesamia inferens] | 67.37 | 62.90 | 1057.26 | 247.90 |
| MsepCSP8 | CL1786.Contig6 | 889 | 128 | Complete ORF | Y | 1e-66 | gb\|AAF71290.2\|AF255919_1 chemosensory protein [Mamestra brassicae] | 1006.17 | 838.60 | 5229.50 | 1939.06 |
| MsepCSP9 | CL5692.Contig4 | 1475 | 127 | Complete ORF | Y | 7e-73 | gb\|AGR39576.1\| chemosensory protein 6 [Agrotis ipsilon] | 520.22 | 699.68 | 504.37 | 1321.90 |
| MsepCSP10 | CL3810.Contig3 | 1019 | 127 | Complete ORF | Y | 1e-62 | gb\|AGY49267.1\| putative chemosensory protein [Sesamia inferens] | 33.03 | 36.16 | 90.50 | 113.15 |
| MsepCSP11 | CL3810.Contig4 | 957 | 127 | Complete ORF | Y | 2e-69 | gb\|AGY49267.1\| putative chemosensory protein [Sesamia inferens] | 28.55 | 24.93 | 84.29 | 34.84 |
| MsepCSP12 | Unigene18561 | 555 | 125 | Complete ORF | Y | 1e-42 | gb\|AGR39571.1\| chemosensory protein 1 [Agrotis ipsilon] | 739.76 | 764.35 | 31036.36 | 842.97 |
| MsepCSP13 | Unigene20793 | 614 | 125 | Complete ORF | Y | 2e-26 | dbj\|BAF34357.1\| chemosensory protein9 [Bombyx mori] | 6.24 | 4.39 | 79.31 | 1.96 |
| MsepCSP14 | CL1279.Contig2 | 1017 | 124 | Complete ORF | Y | 2e-65 | gb\|AIW65100.1\| chemosensory protein [Helicoverpa armigera] | 12.25 | 20.31 | 8.99 | 1.75 |
| MsepCSP15 | Unigene21424 | 560 | 122 | Complete ORF | Y | 2e-39 | dbj\|BAG71920.1\| chemosensory protein 12 [Papilio xuthus] | 6.02 | 6.96 | 14.15 | 3.37 |
| MsepCSP16 | CL3804.Contig2 | 812 | 122 | Complete ORF | Y | 1e-75 | gb\|AFR92094.1\| chemosensory protein 10 [Helicoverpa armigera] | 4.26 | 7.77 | 5.03 | 10.62 |
| MsepCSP17 | Unigene9416 | 704 | 120 | Complete ORF | Y | 2e-63 | gb\|AEX07265.1\| CSP2 [Helicoverpa armigera] | 8959.39 | 6975.81 | 41942.08 | 13202.16 |
| MsepCSP18 | CL8151.Contig1 | 2473 | 111 | Complete ORF | Y | 3e-32 | gb\|AGY49261.1\| putative chemosensory protein [Sesamia inferens] | 3052.75 | 1156.01 | 1609.78 | 2311.30 |
| MsepCSP19 | Unigene38626 | 438 | 111 | Complete ORF | Y | 2e-69 | gb\|AEX07268.1\| CSP7 [Helicoverpa armigera] | 0.00 | 0.07 | 3.33 | 0.00 |
| MsepCSP20 | Unigene21799 | 380 | 110 | 3' lost | Y | 8e-40 | gb\|AII01011.1\| chemosensory protein [Dendrolimus houi] | 2.60 | 0.83 | 0.00 | 1.37 |
| MsepCSP21 | Unigene40442 | 336 | 108 | 5', 3' lost | N | 1e-75 | gb\|AGH20053.1\| chemosensory protein 15, partial [Helicoverpa armigera] | 0.00 | 0.00 | 1.26 | 0.00 |
| MsepCSP22 | Unigene15708 | 801 | 107 | Complete ORF | Y | 6e-58 | gb\|AGR39575.1\| chemosensory protein 5 [Agrotis ipsilon] | 28.93 | 20.10 | 7.11 | 18.22 |
| MsepCSP23 | CL9538.Contig2 | 1363 | 106 | Complete ORF | Y | 4e-47 | gb\|AII01044.1\| chemosensory protein [Dendrolimus kikuchii] | 2.51 | 2.65 | 0.45 | 0.38 |
| MsepCSP24 | Unigene38955 | 370 | 97 | 5' lost | N | 8e-67 | gb\|AFR92095.1\| chemosensory protein 11 [Helicoverpa armigera] | 0.00 | 0.00 | 16.19 | 0.00 |
| MsepCSP25 | Unigene38740 | 257 | 73 | 3' lost | Y | 1e-37 | gb\|AIW65099.1\| chemosensory protein [Helicoverpa armigera] | 0.00 | 0.00 | 3.20 | 0.00 |
| MsepCSP26 | Unigene40591 | 225 | 64 | 3' lost | Y | 6e-40 | gb\|AGZ04932.1\| chemosensory protein 4 [Laodelphax striatella] | 0.00 | 0.00 | 1.30 | 0.00 |

Note: FA: Female antennae; MA: Male antennae; P: Proboscis; LP: Labial palp.

Table S2-6. Unigenes of candidate sensory neuron membrane proteins in *M. separate*

| Gene name | Unigene reference | Length (nt) | ORF (aa) | Status | TMD (No.) | Evalue | BLASTx best hit | FA  FPKM | MA FPKM | P  FPKM | LP FPKM |
| --- | --- | --- | --- | --- | --- | --- | --- | --- | --- | --- | --- |
| MsepSNMP1 | Unigene26386 | 2179 | 525 | Complete ORF | 2 | 0 | sp\|Q8I9S1.1\|SNMP1_HELAM RecName: Full=Sensory neuron membrane protein 1 [Helicoverpa armigera] | 260.94 | 367.60 | 3.28 | 4.08 |
| MsepSNMP2 | CL8696.Contig1 | 2517 | 520 | Complete ORF | 2 | 0 | gb\|AGF87120.1\| sensory neuron membrane protein 2 [Agrotis ipsilon] | 22.37 | 15.23 | 8.24 | 3.08 |

Note: FA: Female antennae; MA: Male antennae; P: Proboscis; LP: Labial palp.
